# Transport oil product consumption and GHG emission reduction potential in China: An electric vehicle-based scenario analysis

**DOI:** 10.1371/journal.pone.0222448

**Published:** 2019-09-16

**Authors:** Yuhua Zheng, Shiqi Li, Shuangshuang Xu

**Affiliations:** School of Economics and Management, China University of Petroleum-Beijing, Beijing, China; Nanyang Technological University, SINGAPORE

## Abstract

China’s transport sector is facing enormous challenges from soaring energy consumption and greenhouse gas (GHG) emissions. Transport electrification has been viewed as a major solution to transportation decarbonization, and electric vehicles (EVs) have attracted considerable attention from policymakers. This paper analyzes the effects of the introduction of EVs in China. A system dynamics model is developed and applied to assess the energy-saving and emission-reducing impacts of the projected penetration of EVs until the year 2030. Five types of scenarios of various EV penetration rates, electricity generation mixes, and the speed of technological improvement are discussed. Results confirm that reductions in transport GHG emissions and gasoline and diesel consumption by 3.0%–16.2%, 4.4%–16.1%, and 15.8%–34.3%, respectively, will be achieved by 2030 under China’s projected EV penetration scenarios. Results also confirm that if EV penetration is accompanied by decarbonized electricity generation, that is, the use of 55% coal by 2030, then total transport GHG emissions will be further reduced by 0.8%–4.4%. Moreover, further reductions of GHG emissions of up to 5.6% could be achieved through technological improvement. The promotion of EVs could substantially affect the reduction of transport GHG emissions in China, despite the uncertainty of the influence intensity, which is dependent on the penetration rate of EVs, the decarbonization of the power sector, and the technological improvement efficiency of EVs and internal combustion engine vehicles.

## 1 Introduction

The transport sector plays a key role in energy consumption and greenhouse gas (GHG) emissions. Approximately 19% of energy consumption and 50% of oil consumption in the world are related to transportation. Moreover, and 23% of CO_2_ emissions are likewise derived from various modes of transportation [1]. In 2016, CO_2_ emissions from the transport sector exceeded CO_2_ emissions from electricity generation for the first time in 38 years, thereby becoming the largest source of carbon emissions [2–3]. Energy consumption in China’s transport sector reached 396.5 million tons of coal equivalents in 2017 [3], which accounted for 9.1% of the total energy consumption. However, in developed countries generally account for 25%–30%. In the same year, the GHG emissions in China’s transport sector accounted for 8.9% of total emissions (specifically, 31.7% and 17.9%in the United States and Japan, respectively). China’ transport sector has seen a dramatic growth in recent years. Transportation passengers and freight turnovers have increased nearly 10 times since 1980. In addition, the motor vehicle stock has increased by 14% annually. However, vehicle ownership per thousand people is only 15% of that in the United States (766 per 1,000 people) [4]. Given the development of the economy, the continuous urbanization process, and the increase in per capita income, China’s transport demand is expected to increase rapidly in the coming decades. Compared with other sectors, the transport sector is facing enormous pressure from soaring energy consumption and GHG emissions. In addition, the study [5] claimed that traffic GHG emissions may be underestimated because GHG emissions from nontraffic social vehicles are excluded. From 2000 to 2017, the intensity of China’s general energy and industrial energy consumption decreased by 59%, and 53%, respectively. However, the intensity of transport energy consumption decreased by only 34% [6]. Moreover, GHG emissions in the transportation process are difficult to capture and seal. Therefore, the state government has placed considerable importance on the implementation of GHG emission reduction policies in the transport sector.

Transport electrification has been viewed as a major solution to transportation decarbonization [7–8]. Numerous governments have announced their desire to eventually ban cars powered by internal combustion engines. China is likewise ambitious in promoting electric vehicles (EVs). In 2009, the “Automobile Industry Adjustment and Revitalization Plan” was put forward by the State Council with the strategy to promote new energy vehicles for the first time. Only 7,200 new energy vehicles were sold in 2010. However, the new energy vehicle industry completed the initial phase in the “The 12th Five-Year Plan,” with support from national policies. By 2017, the sales volume of new energy vehicles reached 556,000, which indicated an increase of nearly 77 times. Out of this volume, the sales of pure EVs reached 445,000, which accounted for 80% of the total sales. The International Energy Agency (IEA) contended that China’s EV stock in 2016 surpassed that of the United States for the first time [9]. In addition, China’s EV sales in 2017 accounted for more than 40% of the world’s total sales, which was two times more than that of the United States, thereby accounting for approximately one-third of the world’s total stock. Therefore, China is becoming the world’s largest EV country. Consequently, further studies are required to investigate into the effects of EVs on transport fuel saving and emission reduction owing to the increasing GHG emissions from transport fuel consumption and the new trend in transport electrification in China.

The reminder of the paper is structured as follows: Section 2 presents the literature review on existing studies of the environmental effects of EVs. Section 3 constructs the system dynamics (SD) model of China’s transport energy consumption and the GHG emission reduction system. Section 4 designs the business as usual (BAU) scenario and other hypothetical EV replacement scenarios in terms of the electricity generation mix and the rate of technological improvement. Sector 5 presents and discusses the simulation results of the various scenarios and compares them with the results of other studies. Finally, Section 6 provides the key conclusions and policy implications.

## 2 Literature review

Previous studies have been conducted on national or regional transport GHG emission reduction policies, in Europe [10–11], the United States [12], Malaysia [13–14], Thailand [15], and China [16–17]. These policies mainly include fuel efficiency improvements, fuel switching, technological improvement, the removal of fuel price subsidy, and public transportation promotion. According to previous research, technological improvement, alternative fuels for vehicles and the removal of fuel price subsidies have the most remarkable effects on the reduction of GHG emissions. However, Huo [16] and Yin et al. [17] claimed that compared with other sectors, China’s transport sector is insensitive to carbon prices. Thus, finding a substitute for high carbon intensive fuels is of considerable importance.

EVs include hybrid EVs (HEVs), plug-in hybrid EVs (PHEVs), and full battery EVs (BEVs). BEVs do not consume gasoline or diesel and are generally verified to have low GHG emissions. Ahmadi [18] proved that BEVs were the most environmentally friendly among EVs in terms of GHG emissions. That is, BEVs released approximately half the amount of emissions by internal combustion engine vehicles (ICEVs). Trost et al. [19] argued that in the medium term, HEVs would dominate the electric powertrain. However, BEVs would dominate in the long-term with the battery technology improvement. PHEVs were also viewed as a promising alternative for reducing emissions owing to its battery packs, which are smaller, compared with BEVs [18] and to improvements in fuel economy through proper energy management [20–23]. The selection of EVs is a complex issue that is dependent on factors such as policy direction, infrastructures, and life cycle costs. Several studies have investigated the environmental effects of the replacement of ICEVs with EVs in China, and the majority agreed that EVs were capable of reducing GHG emissions [24–27].

However, the penetration rate of EVs and regional power generation structures are considered as the two most important factors in transport electrification. These two factors have a considerable impact on the decarbonization of the transport sector [7, 28–29]. The reduction potential of countries such as China, India, and Australia (which rely heavily on coal power) is uncertain and dependent on the carbon intensity of the power generation mix [30–34]. In addition, the technical progress of EVs will also affect the policy implementation of emission reductions [29, 33, 35].

EVs are identified as “zero tailpipe emission” vehicles and gaining popularity around the world. However, the total environmental impacts of EVs have not been fully researched in China. This study intends to analyze the effects of the introduction of EVs in China. Moreover, this study contributes to three main perspectives. First, a systematic framework is proposed to assess the impacts of EVs on China’s transportation energy consumption and on the reduction of GHG emissions. The comprehensive framework combines a macro-energy–economy–environment system with micro-transport–technological selections. Second, the proposed framework is employed to assess the environmental impact of the diffusion of EVs under projected scenarios of various EV penetration rates. Finally, we simulate and discuss the role of China’s electricity grid mix decarbonization as well as the technological improvement of ICEVs and EVs on the reduction of transport GHG emissions.

## 3 Methods and data sources

### 3.1 SD model of CTEGER

**Fig 1:**
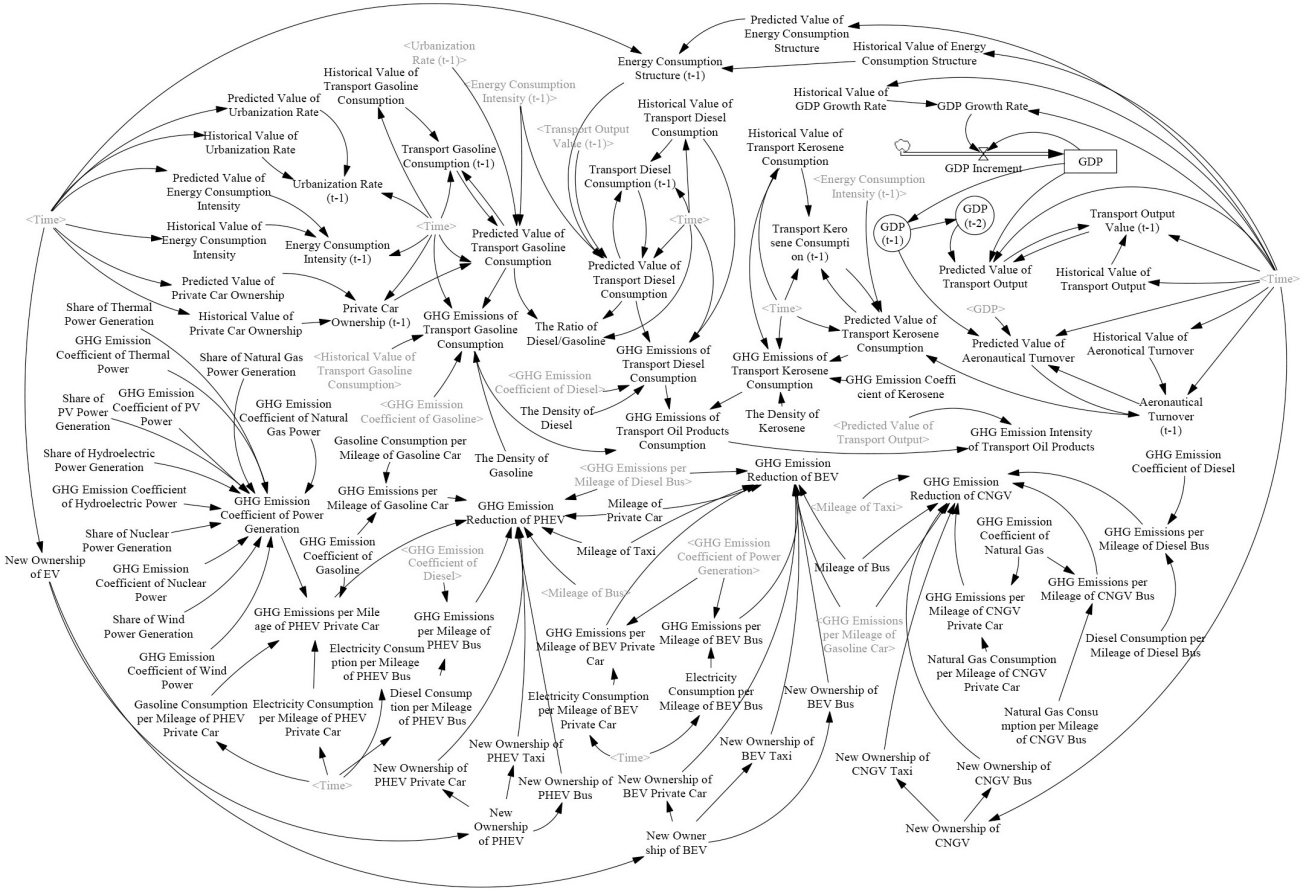
CTEGER.

The SD model is popular in the fields of energy system simulation and policy analysis because of its merits in linking the macro-level energy system with the micro-level structure. Thus, the China’s transportation energy consumption and GHG emission reduction (CTEGER) model is constructed by using the SD method (Fig 1). This system depicts transport energy consumption (e.g., gasoline, diesel, kerosene, natural gas and electricity), the influencing factors that affect it, and GHG emissions. In this system, the quantitative feedback relationship between every two variables is determined by mathematical equations. More than 90 equations exist in this model to express the quantitative relationships between the parameters and the variables. The S1 Appendix provides the specific equations. The simulation period of the SD model ranges from 1985 to 2030 and is divided into two stages. The first stage is for the period of 1985 to 2016, which is used to debug and test the accuracy of the model. The second stage is for the period of 2017 to 2030, which is used for the forecast and policy analysis. The SD model of CTEGER includes two major subsystems, namely, transport oil products consumption and GHG emission subsystem and EV and GHG emission reduction subsystems. The former is a macro-level system, and the latter a micro-level system.

#### 3.1.1 Transport oil products consumption and GHG emission subsystem

**Fig 2:**
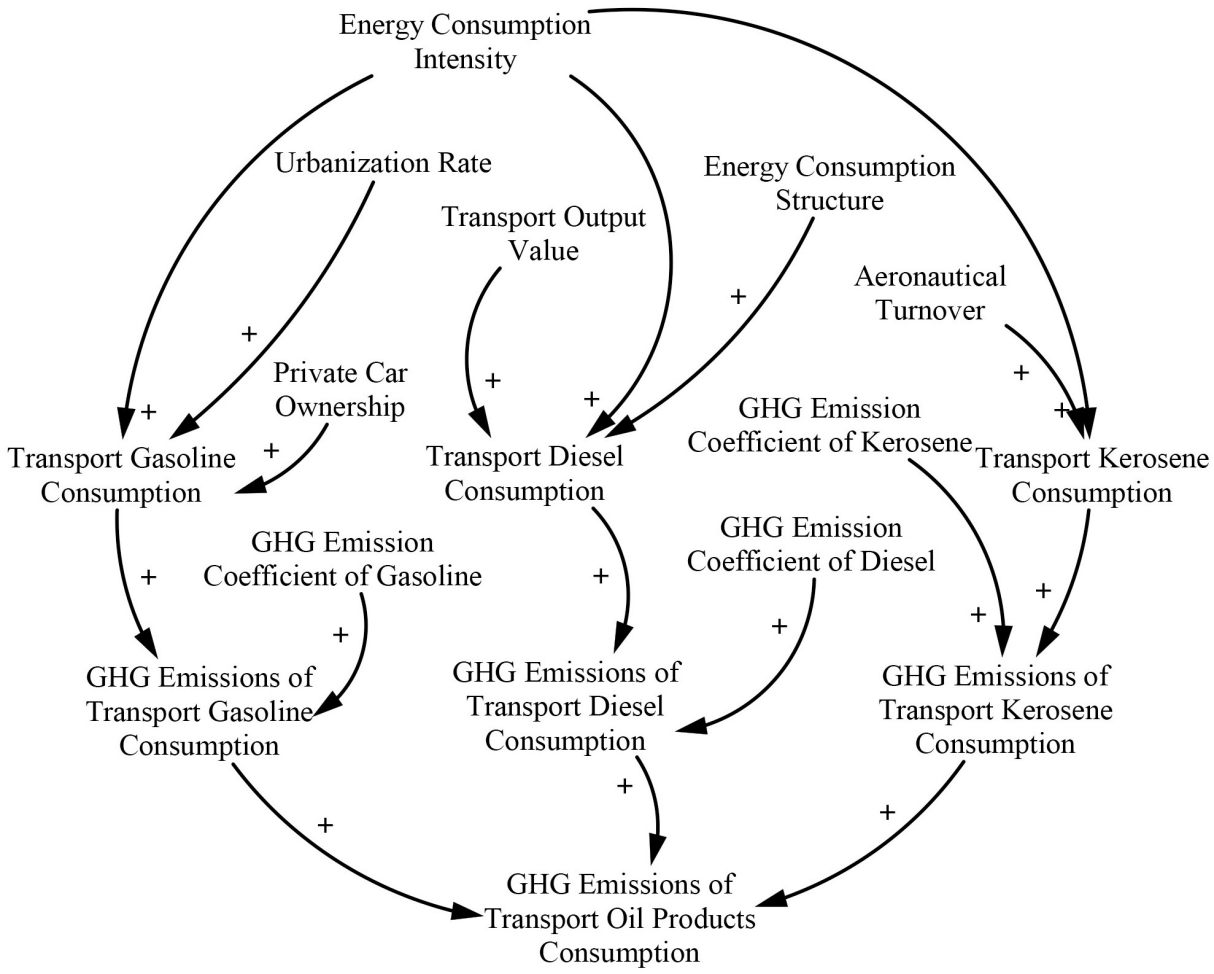
Transport oil product consumption and GHG emission subsystem.

The transport oil products consumption and GHG emission subsystem describe the relationships between the various influencing factors and the types of oil product consumption (Fig 2). Oil products such as gasoline, diesel, and kerosene are the main sources of fuels used in transportation. In 2017, the consumption of these types of oil products accounted for 84% of the total transport energy consumption. The remainder is mainly composed of natural gas (7.5%) and electricity (3.4%) [36]. Several studies have revealed the influencing factors that affect transport energy consumption [37–43], such as economic development, income growth, urbanization, transport energy structure, and fuel efficiency. Referencing these analyses, long-term cointegration relationships between the consumption of gasoline, diesel, and kerosene as well as its influencing factors are established in Eqs (1)–(3) by using step regression. These equations serve as the main relationships among variables to build the subsystem, as shown in Fig 2.

$$\text{ln}\;TG=4.653\;\text{ln}\;UR+0.655\;\text{ln}\;PC+3.836\;\text{ln}\;EI-17.498+e_{t}$$ (1)

$$\text{ln}\;TD=0.877\;\text{ln}\;EI+1.298\;\text{ln}\;ES+1.283\;\text{ln}\;TO-8.754+e_{t}$$ (2)

$$\text{ln}\;TK=1.322\;\text{ln}\;AT+2.387\;\text{ln}\;EI-3.420+e_{t}$$ (3)

where *TG*, *TD*, and *TK* are transport gasoline, diesel, and kerosene consumption, respectively (10^4^ tons); *UR* refers to the economic urbanization rate, which is the ratio of nonagricultural output value to GDP (%); *PC* is private car ownership (10^4^); *EI* is energy consumption intensity (tce/10^4^ yuan); *ES* represents the energy consumption structure, which is the proportion of coal in primary energy consumption (%); *TO* is the transport output value (10^8^ yuan); and *AT* is the aeronautical passenger turnover (10^8^ passenger kilometers). Table 1 summarizes the coefficients of the cointegration equations and the results of the *t*-test. The stationary test also shows that the residual sequences of the cointegration equations of gasoline, diesel, and kerosene remain stable. Thus, all equations are valid.

**Table 1:** Results of cointegration and ECM equations.

	Gasoline		Diesel		Kerosene	
	Variables	Coefficient	Variables	Coefficient	Variables	Coefficient
Cointegration Eqs (1)~(3)	Ln*UR*	4.653* ^b^	ln*EI*	0.877*** ^b^	ln*AT*	1.322***
	ln*PC*	0.655***	ln*ES*	1.298*	ln*EI*	2.387***
	ln*EI*	3.836***	ln*TO*	1.283***		
	*c* ^a^	-17.498** ^b^	*c*	-8.754**	*c*	-3.420***
ECM Eqs (4)~(6)	⊿ln*TG*_*t*-3_	-0.264	⊿ln*TD*_*t*-1_	0.452**	⊿ln*TK*_*t*-1_	-0.207
	⊿ln*EI*_t-1_	0.726	⊿ln*TD*_*t*-3_	0.285	⊿ln*AT*_*t*-1_	0.200*
	⊿ln*EI*_*t*-2_	-0.975	⊿ln*EI*_*t*-3_	-0.413	⊿ln*EI*_*t*-1_	-0.420
	⊿ln*EI*_*t*-3_	0.658	⊿ln*TO*_*t*-3_	0.404		
	⊿ln*TG*_*t*-1_	-0.209	⊿ln*ES*_*t*-1_	1.167*		
	⊿ln*PC*_*t*-3_	0.419				
	⊿ln*UR*_*t*-1_	-2.259*				
	⊿ln*UR*_*t*-2_	-0.930				
	*ecm* (-1)^a^	-0.065**	*ecm* (-1)	-0.079*	*ecm* (-1)	-0.155**
	*c*	0.218**	*c*	0.047	*c*	-0.204***

Note:

^a^ *ecm*(-1) denotes the error correction coefficient;

*c* denotes the constant term.

^b^ *denotes 10% significant level,

**denotes 5% significant level,

***denotes 1% significant level.

The cointegration equation describes the long-term relationship between oil product consumption and economic variables. However, it fails to reflect the characteristics of short-term dynamic changes. We establish error correction models (ECMs) to describe the short-term dynamic relationship among the variables. We use the residual error of the model as the nonequilibrium error after combining the Akaike information and Schwarz criteria. Eqs (4)–(6) show the ECM equations of three kinds of oil product consumption as well as their influencing factors. The stability tests of the ECM models are performed, and the results confirm that the reciprocal eigenvalues of the three equations are less than 1. This finding indicates the validity of the ECMs. Eqs (4)–(6) serve as key causal relationships in the subsystem (see Fig 2). The coefficients of the ECM equations and the result of the *t*-test are shown in Table 1.

$$\begin{array}{ll} \Delta \text{ln}\;TG_{t}= & -0.065\times (\text{ln}\;TG_{t-1}-3.836\;\text{ln}\;EI_{t-1}-0.655\;\text{ln}\;PC_{t-1}-4.653\;\text{ln}\;UR_{t-1}^{}\\ & +17.498)-0.264\Delta \text{ln}\;TG_{t-3}+0.726\Delta \text{ln}\;EI_{t-1}-0.975\Delta \text{ln}\;EI_{t-2}+0.658\Delta \text{ln}\;EI_{t-3}\\ & -0.209\Delta \text{ln}\;TG_{t-1}+0.419\Delta \text{ln}\;PC_{t-3}-2.259\Delta \text{ln}\;UR_{t-1}-0.930\Delta \text{ln}\;UR_{t-2}+0.218 \end{array}$$ (4)

$$\begin{array}{ll} \Delta \text{ln}\;TD_{t}= & -0.079\times (\text{ln}\;TD_{t-1}-0.877\;\text{ln}\;EI_{t-1}^{}-1.298\;\text{ln}\;ES_{t-1}\\ & -1.283\;\text{ln}\;TO_{t-1}+8.754)+0.452\Delta \text{ln}\;TD_{t-1}+0.285\Delta \text{ln}\;TD_{t-3}\\ & -0.413\Delta \text{ln}\;EI_{t-3}-0.404\Delta \text{ln}\;TO_{t-3}+1.167\Delta \text{ln}\;ES_{t-1}+0.047 \end{array}$$ (5)

$$\begin{array}{ll} \Delta \text{ln}\;TK_{t}= & -0.155\times (\text{ln}\;TK_{t-1}-1.322\;\text{ln}\;AT_{t-1}^{}-2.387\;\text{ln}\;EI_{t-1}+3.420)\\ & -0.207\Delta \text{ln}\;TK_{t-1}+0.200\Delta \text{ln}\;AT_{t-1}-0.420\Delta \text{ln}\;EI_{t-1}+0.204 \end{array}$$ (6)

#### 3.1.2 EV and GHG emission reduction subsystem

**Fig 3:**
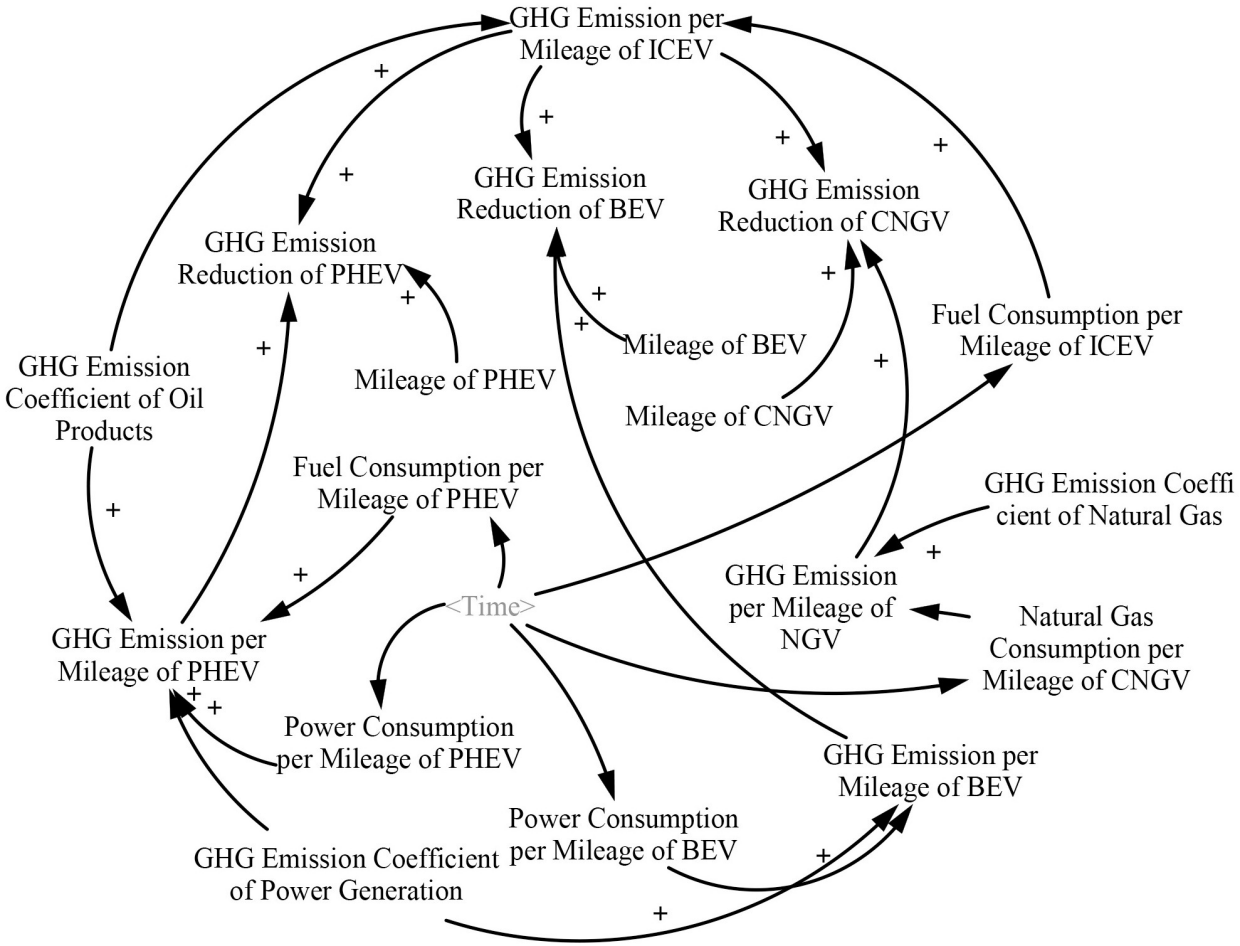
EV and GHG emission reduction subsystem.

Although EVs do not generate direct GHG emissions, they are responsible for indirect emissions discharged from electricity generation. Thus, the EV and GHG emission reduction subsystem (Fig 3) is built to simulate the replacement of ICEVs with BEVs and PHEVs (which are the main research focus in China). Moreover, the subsystem simulates oil product consumption and the reduction of GHG emissions in the transport sector under different scenarios. The GHG emission reduction effect of BEVs and PHEVs is dependent on the difference between the energy consumed per mileage and the GHG emission coefficients of different energy sources.

GHG emissions from the consumption of oil products are obtained on the basis of the life cycle emission coefficient in this subsystem. Two methods are used to measure GHG emissions from fossil fuel consumption according to the Intergovernmental Panel on Climate Change (IPCC) [44], namely, the top-down and bottom-up models. The top-down model calculates GHG emissions based on energy consumption and conversion factors. The bottom-up model differentiates between travel modes and vehicle types and uses travel distance and unit fuel consumption to calculate GHG emissions. The bottom-up model of GHG emission analysis is considered suitable only for small regional research [45]. The present study uses the top-down method because it is capable of calculating transport energy subtypes such as gasoline, diesel, and kerosene.

Following the well-to-wheel (WTW) method, this study derives the life cycle GHG emission coefficients (LCGEC) of gasoline, diesel, and kerosene from four processes, namely, extraction, refining, pipeline transportation, and usage, with reference to [44] and [46]. In summary, we obtain the LCGEC of gasoline, diesel, and kerosene at 2,781.57 gCO_2e_/L, 3,827.94 gCO_2e_/L, and 3,448.80 gCO_2e_/L, respectively. The LCGEC of electricity is also deduced based on the complete life cycle, considering various power generation modes, as shown in Eq (7).

$$\Psi =\sum_{i=1}^{n}\lambda_{i}\theta_{i}$$ (7)

where *Ψ* represents the average LCGEC of electricity generation in China, *λ*_*i*_ represents the proportion of power generation from the *i*-th fuel, and *θ*_*i*_ represents the LCGEC of the *i*-th fuel. The range of LCGEC of different power-generating sources in China and the LCGEC values used in this study are shown in Table 2. According to [6], in 2016, coal-fired power accounts for 68.2% of the electricity generation mix, as shown in Table 2.

**Table 2:** LCGEC of different power-generating sources.

	Share in the electricity generation mix (in year 2016)	Range of LCGEC in China (gCO_2e_/kWh)	LCGEC used in this study (gCO_2e_/kWh)
Coal-fired power	68.2%	900–1342 (Refs. [46–50])	1133
Natural gas-fired power	4%	573–973 (Refs. [49–51])	773
Photovoltaic Power	1%	20–240 (Refs. [49–51])	140
Hydropower	19.4%	1–50 (Refs. [49–53])	17
Nuclear power	3.5%	6.2–11.9 (Refs. [49–51, 54])	9
Wind power	3.9%	6.5 −68 (Refs. [49–51, 55])	32

### 3.2 Data sources and model test

#### 3.2.1 Data sources

Data on the oil product consumption of gasoline, diesel, and kerosene as well as other variables are collected from Refs. [3, 6]. However, the definition and coverage of transport oil product consumption differ among bodies of research. For example, in Refs. [3, 6], oil products consumed by the transportation, storage, and post sectors only cover the fuel consumption of commercial vehicles. However, the consumption of other transportation oil products is assigned to the residential, commercial, and industrial sectors. We use the allocation method by Wang (2010) [56] to estimate total transport oil product consumption, which is currently widely referred to by various scholars [17]. Wang suggests that in addition to oil products consumed by the transportation, storage, and post sectors, 95% of gasoline and 35% of diesel used in the industry and commercial sectors as well as all gasoline and 95% of diesel used in the residential and agricultural sectors are allocated to transportation.

GHGs emitted during the production and consumption of oil products and electricity are expressed as CO_2_ equivalent, according to the global warming potential (GWP) from the IPCC Assessment Report [44]. For instance, 1 gCH_4_ has a GWP value of 25 gCO_2e_, whereas 1 gN_2_O has a GWP value of 298 gCO_2e_.

#### 3.2.2 Model test

Variables in the CTEGER SD model are evaluated in this paper by historical and operating tests.

**Fig 4:**
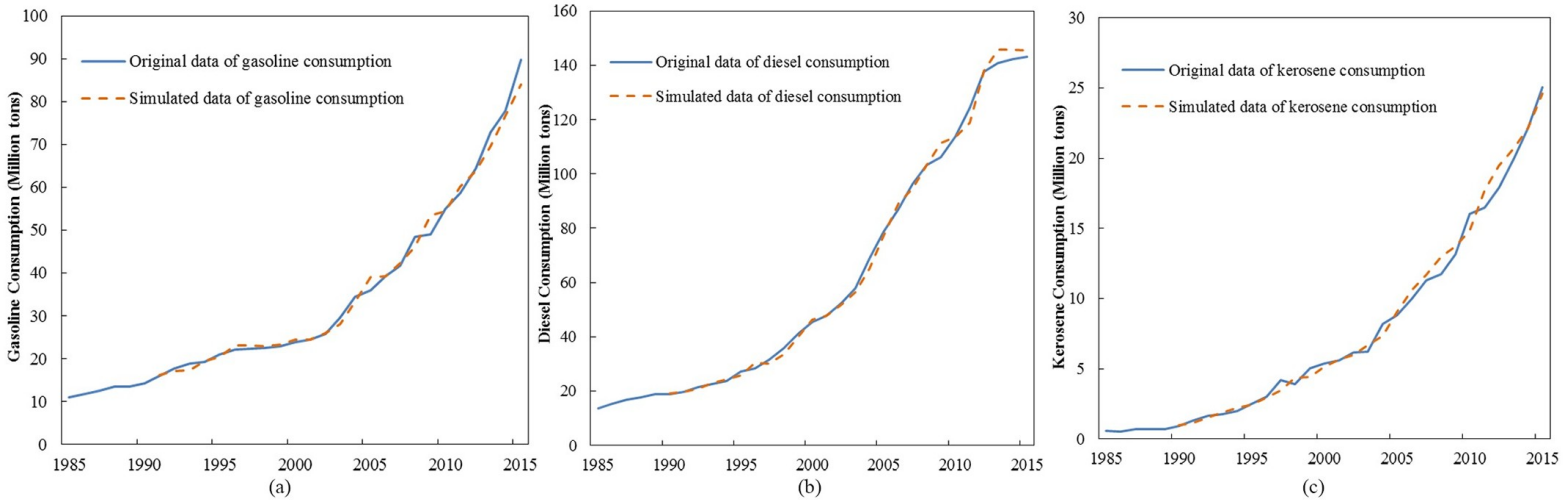
Results of historical tests. (a) gasoline consumption and (b) diesel consumption and (c) kerosene consumption.

The historical test refers to the evaluation of the fitting degree between the simulated results of the model and original data to verify the validity of the system model. That is, the smaller the relative error between the simulated and original data, the better the simulation effect of the system model and the more convincing the prediction value. Variables in the model are verified by using the historical test to determine if the CTEGER model is consistent with reality. This test involves the *TO*, *UR*, *PC*, *AT*, *TG*, *TD*, and *TK* variables. The historical tests show the following results (Table 3 and Fig 4): 1) For *TO* and *UR*, more than 95% of the errors between the simulated and actual values can be controlled within 5%. 2) For *TG*, *TK*, *AT*, and *PC*, more than 90% of the errors between the simulated and actual values can be controlled within 10%. 3) For *TD*, more than 90% of the errors between the simulated and actual values can be controlled within 5%. These results indicate that the CTEGER model can meet the requirements of the historical test and is consistent with reality. Fig 4(a)–4(c) show the original and simulated series of gasoline, diesel, and kerosene consumption, which are the key variables in CTEGER.

**Table 3:** Summary of historical and operating test errors.

Variable	Error confidence	Range of relative error		
		Historical test error	Operating test error (DT = 1/DT = 0.5)	Operating test error (DT = 1/DT = 0.25)
*TG*	100%–95%		≦3%	≦5%
	95%–90%	≦10%		
*TD*	100%–95%		≦5%	≦5%
	95%–90%	≦5%		
*TK*	100%–95%		≦5%	≦5%
	95%–90%	≦10%		
*TO*	100%–95%	≦5%	≦1%	≦1%
	95%–90%			
*AT*	100%–95%		≦10%	≦10%
	95%–90%	≦10%		
*UR*	100%–95%	≦3%	≦1%	≦1%
	95%–90%			
*PC*	100%–95%		≦1%	≦1%
	95%–90%	≦10%		

**Fig 5:**
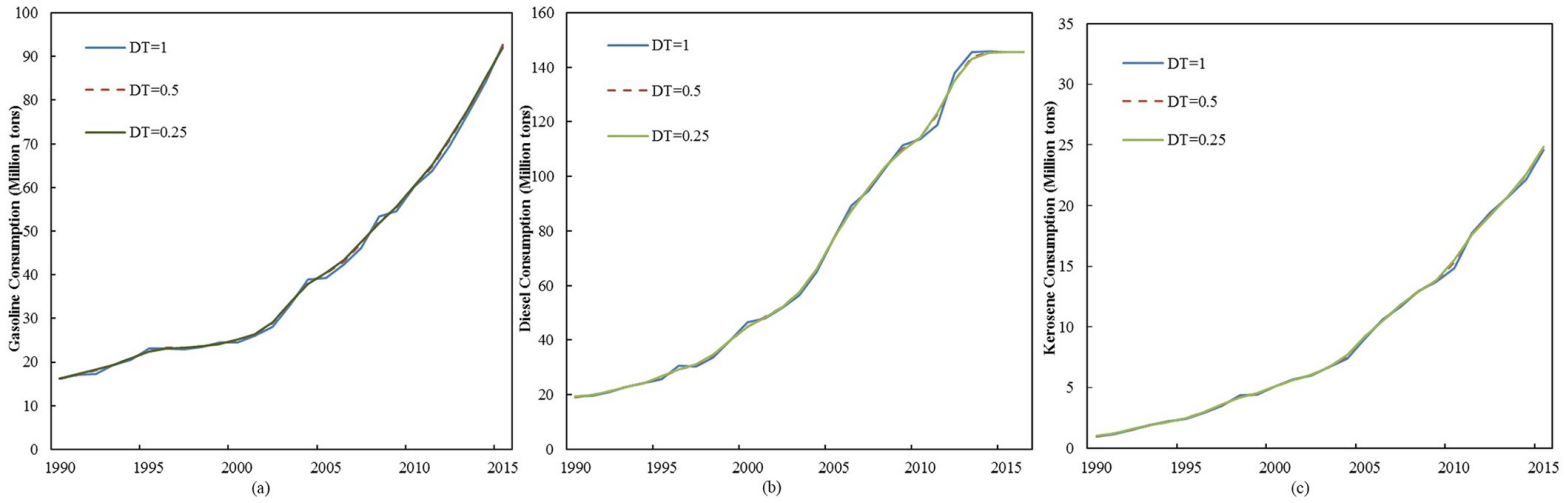
Results of operating tests. (a) gasoline consumption and (b) diesel consumption and (c) kerosene consumption.

The operating test is used to verify the correctness of the simulation model as well as to determine its rationality and stability. The simulation step (denoted as DT, which represents the time interval of data) in the original model is 1 (i.e., 1 year as the interval). Then, the model is tested on the condition of DT = 0.5 and DT = 0.25. The simulation results (Fig 5) show that the three curves of DT = 1, DT = 0.5, and DT = 0.25 are basically coincident with one another. This finding implies that the system is stable. Table 4 presents the relative errors of the operating test results, which show the error between DT = 1 and DT = 0.5 as well as between DT = 1 and DT = 0.25.

**Table 4:** Descriptions and assumptions of scenarios.

Scenario Type	Scenario Name	Descriptions and Assumptions
BAU	BAU	Oil product consumption and GHG emissions are predicted based on Eqs (4)–(6) without further policy measures to promote the development of EVs.
EV replacement	EVH	EV high (EVH) replacement scenario assumes that EV ownership will increase to 80 million in 2030.
	EVL	EV low (EVL) replacement scenario assumes that EV ownership will increase to 15 million in 2030.
	EVB	EV baseline (EVB) replacement scenario assumes that EV ownership will increase to 47.5 million in 2030.
Decarbonized electricity generation	EVHS	EVH scenario combined with decarbonized electricity generation scenario, which assumes the use of 55% coal in 2030.
	EVLS	EVL scenario combined with decarbonized electricity generation scenario, which assumes the use of 55% coal in 2030.
	EVBS	EVB scenario combined with decarbonized electricity generation scenario, which assumes the use of 55% coal in 2030.
Technological improvement in EVs	EVB+HT (EVBS+HT)	EVB (EVBS) scenario combined with high technological improvement speed of EV, that is, electricity consumption rate of EVs will be reduced by 10% every 5 years until 2030.
	EVB+LT (EVBS+LT)	EVB (EVBS) scenario combined with low technological improvement speed of EV, that is, electricity consumption rate of EVs will be reduced by 5% every 5 years until 2030.
	EVB+BT (EVBS+BT)	EVB (EVBS) scenario combined with moderate technological improvement speed of EV, that is, electricity consumption rate of EVs will be reduced by 7.5% every 5 years until 2030.
Technological improvement in EVs and ICEVs	EVB (EVBS) +HT+ICEV	EVB (EVBS)+HT scenario combined with technological improvement of ICEV, that is, the average fuel consumption rate of gasoline cars and diesel buses will be reduced to 5.5 L/100 km and 29.1 L/100 km, respectively, in 2030.
	EVB (EVBS) +LT+ICEV	EVB (EVBS)+LT scenario combined with technological improvement of ICEV, that is, the average fuel consumption rate of gasoline cars and diesel buses will be reduced to 5.5 L/100 km and 29.1 L/100 km, respectively, in 2030.
	EVB (EVBS) +BT+ICEV	EVB (EVBS)+BT scenario combined with technological improvement of ICEV, that is, the average fuel consumption rate of gasoline cars and diesel buses will be reduced to 5.5 L/100 km and 29.1 L/100 km, respectively, in 2030.

## 4 Scenario design

Five types of scenarios and their underlying assumptions are developed and presented in Table 4. Section 4.1 introduces the BAU scenario as a reference for comparison. Section 4.2 defines six EV replacement scenarios to explore energy conservation and GHG emission mitigation potentials for the transport sector. Section 4.3 defines six EV and ICEV technological improvement scenarios to explore possible additional GHG emission reduction from technological improvement.

### 4.1 BAU scenario

The BAU scenario is defined according to the current trend without further policy measures to promote the development of EVs during the scenario period. This section presents the assumptions and prediction methods of certain key parameters under the BAU scenario.

#### 4.1.1 Energy consumption structure

Two energy consumption structure scenarios are defined according to China’s “13th Five-year Plan” and Ref. [57], namely, the high- and low-carbon scenarios. Under the high-carbon scenario, the ratio of coal in China’s total energy consumption is 59% and 53% in 2020 and 2030, respectively. Under the low-carbon scenario, the ratio of coal in the total energy consumption is 57% and 43% in 2020 and 2030, respectively. The BAU scenario is the average of the high- and low-carbon scenarios.

#### 4.1.2 Transport output value and aeronautical passenger turnover

The transport output value *(TO*) is an economic time series. Given the inertia of economic activity, the psychological habits of people’s transport demand, and investments in fixed assets in transportation, the previous trend of *TO* is expected to continue in the coming years, thereby leading to the change in the *TO* in relation to its past. Therefore, the autoregressive distributed lag (ADL) model is used in this study to project the *TO*. The ADL equation of the relationship between the *TO* and GDP, with a lag period of 1, is built, tested, and shown in Eq (8).

$$\begin{array}{c} \text{ln}\;TO_{t}=0.931\;\text{ln}\;TO_{t-1}+0.278\;\text{ln}\;GDP_{t}-0.223\;\text{ln}\;GDP_{t-1}\\ \left(0.0000\right)\;\left(0.0111\right)\;\left(0.0488\right) \end{array}$$ (8)

The *p* value of each coefficient is shown in brackets. The adjusted coefficient of determination (R^2^) is 0.989, and the Durbin–Watson (DW) value is 1.782, which indicates that no autocorrelation exists. The White test also proves that no heteroscedasticity exists.

Similarly, the ADL method is used to predict aeronautical passenger turnover (*AT*). Eq (9) shows the ADL equation of *AT* and GDP with R^2^ = 0.997, in which no autocorrelation or heteroscedasticity is found.

$$\begin{array}{c} \text{ln}\;AT_{t}=-5.835+0.406\;\text{ln}\;AT_{t-1}+2.996\;\text{ln}\;GDP_{t}-2.174\;\text{ln}\;GDP_{t-1}\\ \left(0.0005\right)\;\left(0.0083\right)\;\left(0.0001\right)\;\left(0.0007\right) \end{array}$$ (9)

#### 4.1.3 Economic growth

There is a close relationship between economic development and oil consumption in China [58]. The present study also proves that economic growth will affect transportation output value and aeronautical passenger turnover (Eqs (8) and (9)), thus affecting the consumptions of oil products (Eqs (2) and (3)). The Chinese economy is currently shifting from a high-speed boom to a sustainable development, with the growth rate declining over time. The present study defines two GDP growth scenarios according to China’s “13th Five-year Plan” and Ref. [59] in which the BAU scenario is deduced according to the GDP. The first is the optimistic scenario of GDP growth under which the GDP growth rate falls by 0.1% per year from 6.9% in 2017 to 2030. The second is the pessimistic scenario of GDP growth under which the GDP growth rate falls by 0.2% per year in the same period. The GDP growth rate under the BAU scenario is the average of the optimistic and pessimistic scenarios.

#### 4.1.4 Urbanization rate and private car ownership

The growth curve model is widely employed for middle- and long-term predictions of limited monotonous growth processes. According to the experiences of developed countries, the urbanization rate (*UR*) and private car ownership (*PC*) undergo a process of beginning, rapid growth, slow growth, and eventually plateau. This process is consistent with the growth curve rule [60–61]. Commonly used growth curves include the logistic curve, the simple S curve, and the Compertz curve. The present study uses the logistic curve (Eq (10)) to project the *UR* and *PC* in China.

$$y=\frac{k}{1+Ae^{-Bt}}$$ (10)

where *y* represents the predicted *UR* or *PC*, *t* denotes different years, *A* and *B* are parameters, and *k* represents the saturation level. In estimating China’s *UR*, the initial value of *k* is assumed to be 95%, with the constraint of the *UR* at less than 100%. The parameters in Eq (10) for the *UR* are deduced as *A* = 0.365 and *B* = 0.678 by using the nonlinear programming method. In this study, we assume that China’s *PC* peaks at 380 million. The *A* and *B* parameters for the logistic curve of *PC* are 2,216 and −0.232, respectively.

#### 4.1.5 Energy intensity

In Section 2.1.1, Δln*EI* is proven as a stable time series with the trailing autocorrelation coefficient, which conforms to the law of auto regression (AR). In addition, its partial correlation coefficient is truncated after the two orders. Eq (11) shows the AR model for Δln*EI*.

$$\begin{array}{c} \Delta \text{ln}\;EI_{t}=-0.035+1.060\Delta \text{ln}\;EI_{t-1}-0.436\Delta \text{ln}\;EI_{t-2}\\ \left(0.0056\right)\;\left(0.0000\right)\;\left(0.0265\right) \end{array}$$ (11)

The reciprocal root of the formula is 0.53 + 0.39i, which is within the unit circle. The DW value of the equation is 2.025, which indicates that no autocorrelation exists. Moreover, the residual is a white noise sequence. Thus, the model is valid.

### 4.2 EV replacement scenarios

In 2015, the Chinese National Development and Reform Commission issued the “Guide for the Development of Electric Vehicle Charging Infrastructure 2015–2020” [62]. A development target of more than five million EVs in China in 2020 was proposed. Toward the end of 2017, the Society of Automotive Engineers, as entrusted by the Ministry of Industry and Information Technology of China, issued the “Energy Saving and New Energy Vehicle Technology Roadmap 2017” [63]. This report proposed that the volume of EVs in 2030 would be in the range of 15–80 million. Therefore, we design three EV replacement scenarios to assess their influence on oil product consumption and GHG emissions. The EVL scenario assumes that EV ownership will increase to 15 million in 2030, whereas the EVH scenario assumes that EV ownership will increase to 80 million in 2030. The EVB is the average of the low and high scenarios. This scenario assumes an EV ownership of 47.5 million in 2030. We assume in each scenario that no changes or upgrades will occur in China’s electricity generating mix and that 68.2% (the current share) of electricity will continue to be generated by coal in 2030. However, the decarbonization of the electricity sector, that is, the reduction of the coal-fired electricity shares, will be further discussed in Section 5 under the EVHS, EVLS, and EVBS scenarios (Table 4).

BEVs accounted for 76% of the total sales of EVs from 2011 to 2017. Therefore, we suppose that BEVs and PHEVs will retain the ratio of 3:1 in the future. The main usages of EVs are private cars, taxis, buses, and special vehicles for sanitation and logistics purposes. According to our investigation and estimation, the ratio of electric private cars, taxis, buses, and special vehicles for sanitation and logistics purposes will be approximately 43:3:2:2 in the near future. In this study, the annual mileage of private cars, taxis, buses, and special vehicles for sanitation and logistics purposes is assumed to be 18,000, 120,000, 60,000 and 60,000 km, respectively. The fuel consumption rate of private gasoline cars is 7.82 L/100 km and that of diesel buses is 36.39 L/100 km [64]. According to the recent data from the Ministry of Industry and Information Technology of the People’s Republic of China, we calculate that the average power consumption of BEV private cars and buses is 18.73 kWh/100 km and 98 kWh/100 km, respectively. The energy consumption of PHEV private cars and buses is 4.3 L/100 km + 9 kWh/100 km and 17.7 L/100km + 37.1 kWh/100 km, respectively [65].

Geng et al. [66] proved that compressed natural gas vehicles (CNGVs) can perform well in terms of emission reduction where the power grid is mainly coal based. The present study also considers the replacement of ICEVs with CNGVs to comprehensively evaluate oil product consumption and the potential reduction of GHG emissions in the transport sector. We assume that CNGVs in China have a fixed growth rate and will reach 10 and 14 million in 2020 and 2030, respectively. CNGVs are mainly composed of taxis and buses. According to our investigation, the natural gas consumption rate of CNGV taxis and buses is 9 m^3^/100 km and 37.3 m^3^/100 km, respectively. Guo et al. [67] proved that the ratio of CNGV taxis to buses is currently 67 to 33 in China. Therefore, we also use this ratio in this study.

### 4.3 Technological improvement scenarios

We define three scenarios of EV technological improvement according to Ref. [63]. The high technological improvement scenario of EV (EVB+HT) is defined as the reduction of electricity consumption per 100 km by 10% every 5 years until 2030. The low technological improvement scenario of EV (EVB+LT) is defined as the reduction of electricity consumption per 100 km by 5% every 5 years until 2030.

We assume another scenario under which the fuel economy of ICEVs will also be technologically improved. According to Ref. [63], one of the milestones in the future development of China’s ICEV technology is that the fuel consumption of new passenger fuel vehicles will drop to 3.2 L/100 km by 2030 and that of new commercial vehicles will be reduced by 20%. Considering actual driving conditions and the vehicle replacement rate, we assume that the average fuel consumption rate of ICEV gasoline cars and diesel buses will be reduced to 5.5 L/100 km (which is also used in Ref. [31]) and 29.1 L/100 km, respectively, by 2030. The combined technological improvement scenarios of EVs and ICEVs will be further discussed in Section 5 under the EVB+HT+ICEV, EVB+BT+ICEV, and EVB+LT+ICEV scenarios (Table 4).

## 5 Results and discussion

### 5.1 Influencing factors of transport oil product consumption

The present study analyzes the influencing factors that affect the consumption of various oil products in China’s transport sector. Three cointegration equations are employed to establish long-term relationships and elasticity among the variables. According to the long-term equilibrium (Eq (1)), the *UR* increases by 1%, *TG* increases by 4.65%; *EI* increases by 1%, *TG* increases by 3.84%; *PC* increases by 1%, and *TG* increases by 0.66%, from 1985 to 2016, given that other influencing factors remained constant. The *UR* and *EI* are the dominant influencing factors on transport gasoline consumption in China. This result reveals that urbanization has greatly promoted the transport demand and has caused a rapid growth in transport gasoline consumption. China is still in the stage of urbanization. Thus, the process of urbanization and the growth of private car stocks will drive a further increase in gasoline consumption. However, the decline in *EI* will play a role in lowering gasoline consumption if no policy measures are implemented.

According to Eq (2), the ratio of coal in total energy consumption increases by 1%, *TD* increases by 1.30%; the *TO* increases by 1%, *TD* increases by 1.28%; *EI* decreases by 1%, and *TD* decreases by 0.88%, given that other influencing factors remain constant. The long-term equilibrium among diesel consumption and its influencing factors shows that *ES* is a significant influencing factor on China’s diesel consumption. The transportation of coal in China is largely dependent on diesel trucks. For example, 84.4% of the annual freight transportation in the Jing-Jin-Ji region (Refer to the Beijing–Tianjin–Hebei urban agglomeration, which is the political and cultural center of China and an important core area of North China’s economy) relies on diesel trucks, among which trucks for coal transportation account for 85% [68]. The adjustment of the *ES* and the control of coal consumption growth will contribute to the decline of diesel consumption growth in the future.

According to Eq (3), a 1% increase in *AT* will lead to a 1.32% increase in *TK*, whereas a 1% decrease in *EI* will lead to a 2.39% decrease in *TK*, given that other influencing factors remain constant.

The absolute value of the error correction coefficients reflects the adjustment to the deviation of the long-term equilibrium. According to the error correction coefficient of −0.065 from Eq (4), the non-equilibrium state of gasoline consumption can be adjusted back to the equilibrium state by 6.5% when the short-term fluctuation deviates from the long-term equilibrium. The error correction coefficients from Eqs (5) and (6) show that when the short-term volatility of diesel and kerosene consumption deviates from the long-term equilibrium, the imbalance can be adjusted to the equilibrium state by 7.9% and 15.5%, respectively. The short-term fluctuation of *TG*, *TD*, and *TK* consumption comes from two sources. The first is the future development trend of the *UR*, *PC*, *EI*, *ES*, and other endogenous variables. The second is the influence of the deviation from the long-term equilibrium.

At present, the ratio of transport oil product consumption to total crude oil consumption is 46%. We assume that this ratio will remain unchanged until 2030. The replacement of ICEVs with EVs will reduce crude oil demand by 9%–23%, which will play an important role in reducing the pressure on China to import crude oil. Conversely, China’s refineries to date are in the stage of overcapacity. High-cost refineries may be eliminated because of the decreasing growth rate of oil product consumption.

### 5.2 Transport oil product consumption and GHG emissions under the BAU scenario

**Fig 6:**
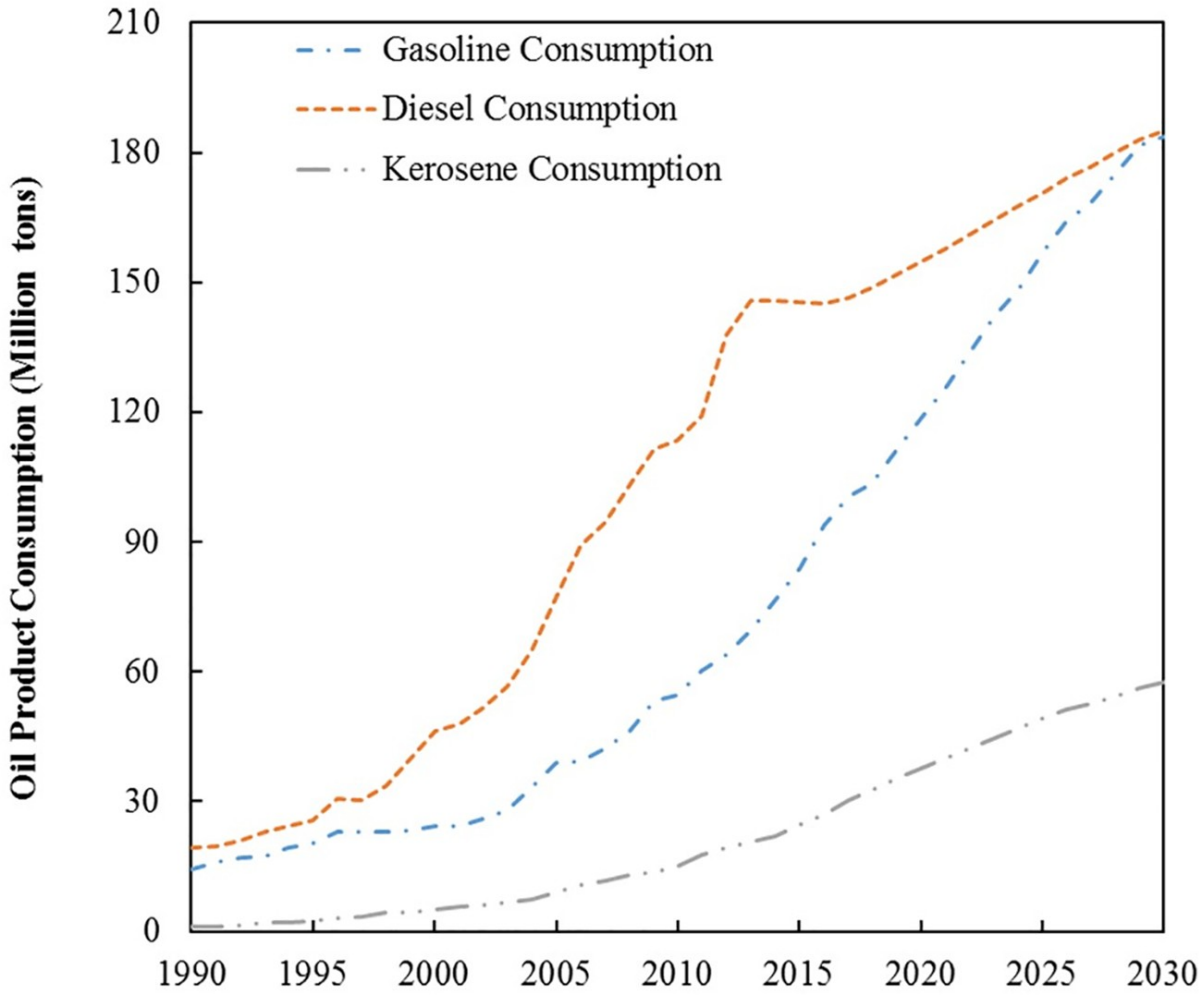
Transport oil product consumption under the BAU scenario.

**Fig 7:**
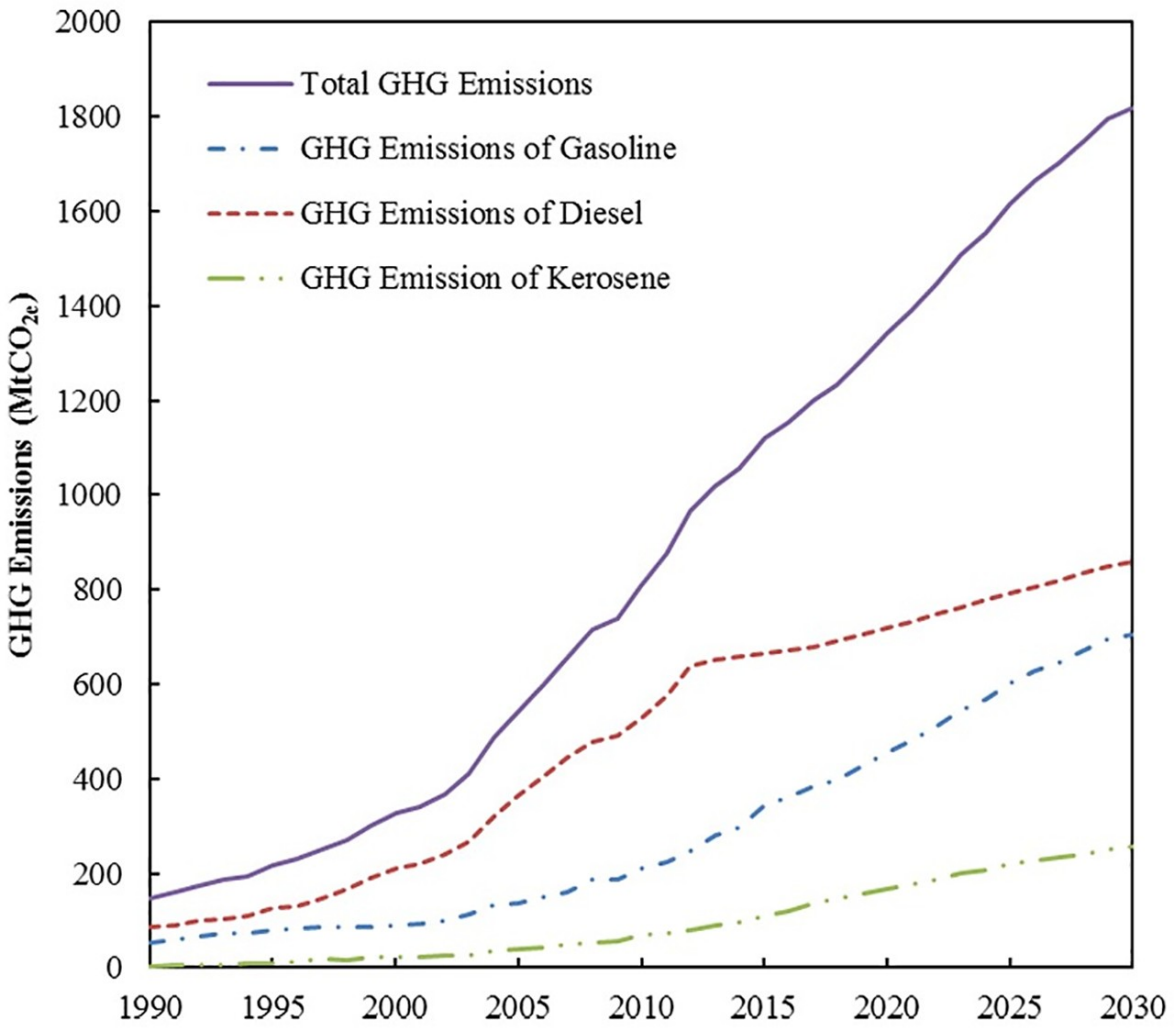
Transport GHG emissions from oil product consumption under the BAU scenario.

Figs 6 and 7 present oil product consumption and GHG emissions from China’s transport sector under the BAU scenario. The simulation results confirm that transport gasoline consumption will maintain an increasing trend, thereby reaching 118.44 Mt and 183.57 Mt in 2020 and 2030, respectively. Transport diesel consumption will increase from 154.74 Mt in 2020 to 185.19 Mt by 2030. However, the annual growth rate of gasoline consumption will decrease from 7.1% in 2020 to 1.1% by 2030. Moreover, the annual growth rate of diesel consumption will decrease from 2.0% in 2020 to 1.2% by 2030. Transport kerosene consumption will increase to 37.66 Mt and 57.48 Mt in 2020 and 2030, respectively. The diesel to gasoline ratio in China’s transport sector will continue to decrease in the simulated time period. According to the experiences of developed countries, when a country enters the late stage of industrialization, the diesel to gasoline ratio declines continuously. China is expected to enter the late stage of industrialization during the “13th Five-Year Plan” period. The ratio reached two peak points, namely, 2.89 and 2.15 in 2006 and 2012, respectively. Thereafter, the present study predicts that the ratio will decrease to 1.35 and 1.00 in 2020 and 2030, respectively, which is in accordance with the development process of developed countries.

GHG emissions from the consumption of gasoline, diesel, and kerosene in the transport sector will increase from 454.41 MtCO_2e_, 717.98 MtCO_2e_, and 167.61 MtCO_2e_ in 2020 to 704.30 MtCO_2e_, 859.28 MtCO_2e_, and 255.81 MtCO_2e_ in 2030, respectively, as presented in Fig 7. GHG emissions from diesel consumption comprise the dominant share all the way through 2030. However, the share will decrease from 56.6% to 47.2% during the simulation period.

### 5.3 GHG emission reduction under various EV replacement scenarios

#### 5.3.1 GHG emission reduction effect of EV under different electricity generation mixes

The CTEGER model is used to simulate the reduction of GHG emissions from the replacement of gasoline and diesel vehicles with BEVs and PHEVs. Column 3 in Table 5 shows the GHG emission coefficient 100-kilometer of ICEVs, EVs, and CNGVs under the present electricity generation mix (68.2% of electricity is generated by coal). The results indicate that the per 100-kilometer GHG emissions of BEV, PHEV, and CNGV private cars is 27.2%, 10.7%, and 8.1%, respectively, less than that of current gasoline cars. The GHG emissions of BEV, PHEV, and CNGV buses are 40.5%, 17.6%, and 40.2%, respectively, less than that of current diesel buses. However, if the share of coal-fired electricity is reduced to 60%, then the per 100-kilometer GHG emissions of BEV cars and buses will decrease by 37.6% and 49.0%, respectively, and that of PHEV cars and buses will decrease by 15.6% and 22.2%, respectively (Column 4 in Table 5). If the share of coal-fired electricity is reduced to 55%, then the per 100-kilometer GHG emissions of BEV cars and buses will decrease by 46.9% and 56.6%, respectively, and that of PHEV cars and buses will decrease by 20.2% and 26.4%, respectively (Column 5 in Table 5).

**Table 5:** Emission coefficient of various types of vehicles.

Vehicle types	Vehicle usages	Emission coefficient (gCO_2e_/100 km)		
		(68.2% coal fired)	(60% coal fired)	(55% coal fired)
Gasoline vehicles	Private cars and taxis	21,752	–	–
Diesel vehicles	Buses	139,299	–	–
BEVs	Private cars and taxis	15,842	13,583	11,541
	Buses	82,891	71,072	60,387
PHEVs	Private cars and taxis	19,434	18,349	17,367
	Buses	114,838	108,407	102,550
CNGVs	Taxis	19,988	–	–
	Buses	83,282	–	–

Data source: Refs. [64–65]

#### 5.3.2 GHG emission reduction from EV replacement

**Fig 8:**
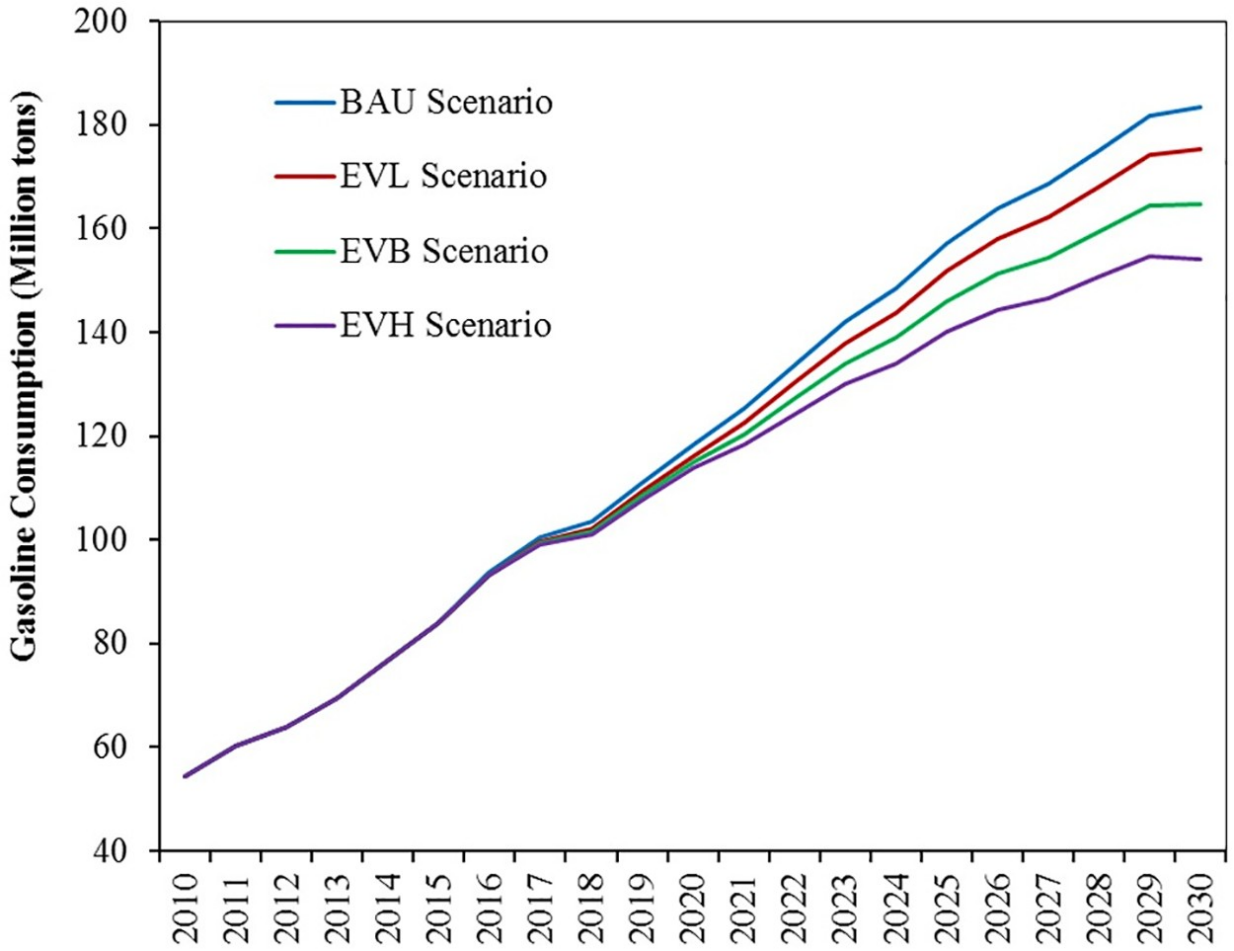
Simulation results of gasoline consumption under different scenarios of EV replacement.

**Reduction in oil product consumption**. The simulation results under the BAU scenario show that total gasoline consumption will be 118.43 Mt in 2020. Gasoline consumption under the EVH, EVB, and EVL scenarios will be 113.92 Mt, 115.05 Mt, and 116.18 Mt, respectively, in 2020. In essence, gasoline consumption will decrease by 3.8%, 2.8%, and 1.9% of the BAU scenario. Total gasoline consumption under the BAU scenario will be 183.57 Mt in 2030. In the same year, gasoline consumption under the EVH, EVB, and EVL scenarios will be 154.01 Mt, 164.71 Mt, and 175.42 Mt, respectively. Thus, gasoline consumption is reduced by 16.1%, 10.3%, and 4.4% of the BAU scenario. In addition, the increase in gasoline consumption will slow down after 2029 under the BAU, EVL, and EVB scenarios. In contrast, gasoline consumption will peak in 2029 under the EVH scenario (Fig 8).

**Fig 9:**
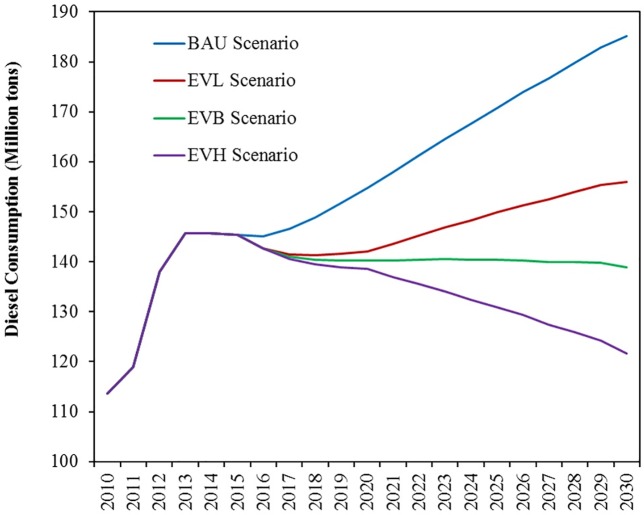
Simulation results of diesel consumption under different scenarios of EV replacement.

The simulation results under the BAU scenario indicate that total diesel consumption will be 154.74 Mt in 2020. The diesel consumption under the EVH, EVB, and EVL scenarios are 138.53 Mt, 140.32 Mt, and 142.13 Mt, respectively. Therefore, diesel consumption is reduced by 10.5%, 9.3%, and 8.1% of the BAU scenario. Total diesel consumption under the BAU scenario will be 185.19 Mt in 2030. In the same year, diesel consumption under the EVH, EVB, and EVL scenarios will be 121.69 Mt, 138.82 Mt, and 155.95 Mt, respectively. Thus, diesel consumption will be reduced by 34.3%, 25.0%, and 15.8% of the BAU scenario (Fig 9). Diesel consumption will slowly increase under the EVL scenario, will remain stable under the EVB scenario, and will steadily decline under the EVH scenario during the simulation period.

**Reduction in GHG emissions**. According to Section 5.2, GHG emissions from oil product consumption under the BAU scenario will be 1,340 MtCO_2e_ in 2020. In the same year, with the increase in BEV ownership, GHG emission reduction from the replacement of ICEVs with BEVs under the EVH, EVB, and EVL scenarios will be 26.42 MtCO_2e_, 15.25 MtCO_2e_, and 4.08 MtCO_2e_, respectively. Therefore, GHG emissions are reduced by 2.0%, 1.1%, and 0.3% of the BAU scenario. GHG emissions from oil product consumption under the BAU scenario will be 1,819.38 MtCO_2e_ in 2030. In the same year, GHG emission reduction from the replacement of ICEVs with BEVs under the EVH, EVB, and EVL scenarios will be 260.22 MtCO_2e_, 154.06 MtCO_2e_, and 47.91 MtCO_2e_, respectively. Thus, GHG emissions are reduced by 14.3%, 8.47%, and 2.6% of the BAU scenario.

In 2020, GHG emission reduction from the replacement of ICEVs with PHEVs under the EVH, EVB, and EVL scenarios will be 3.58 MtCO_2e_, 2.07 MtCO_2e_, and 0.55 MtCO_2e_, respectively. Therefore, GHG emissions are reduced by 0.27%, 0.15%, and 0.19% of the BAU scenario. In 2030, GHG emission reduction from the replacement of ICEVs with PHEVs under the EVH, EVB, and EVL scenarios will be 35.25 MtCO_2e_, 20.87 MtCO_2e_, and 6.49 MtCO_2e_, respectively. Thus, GHG emissions are reduced by 1.94%, 1.15%, and 0.36% of the BAU scenario.

**Fig 10:**
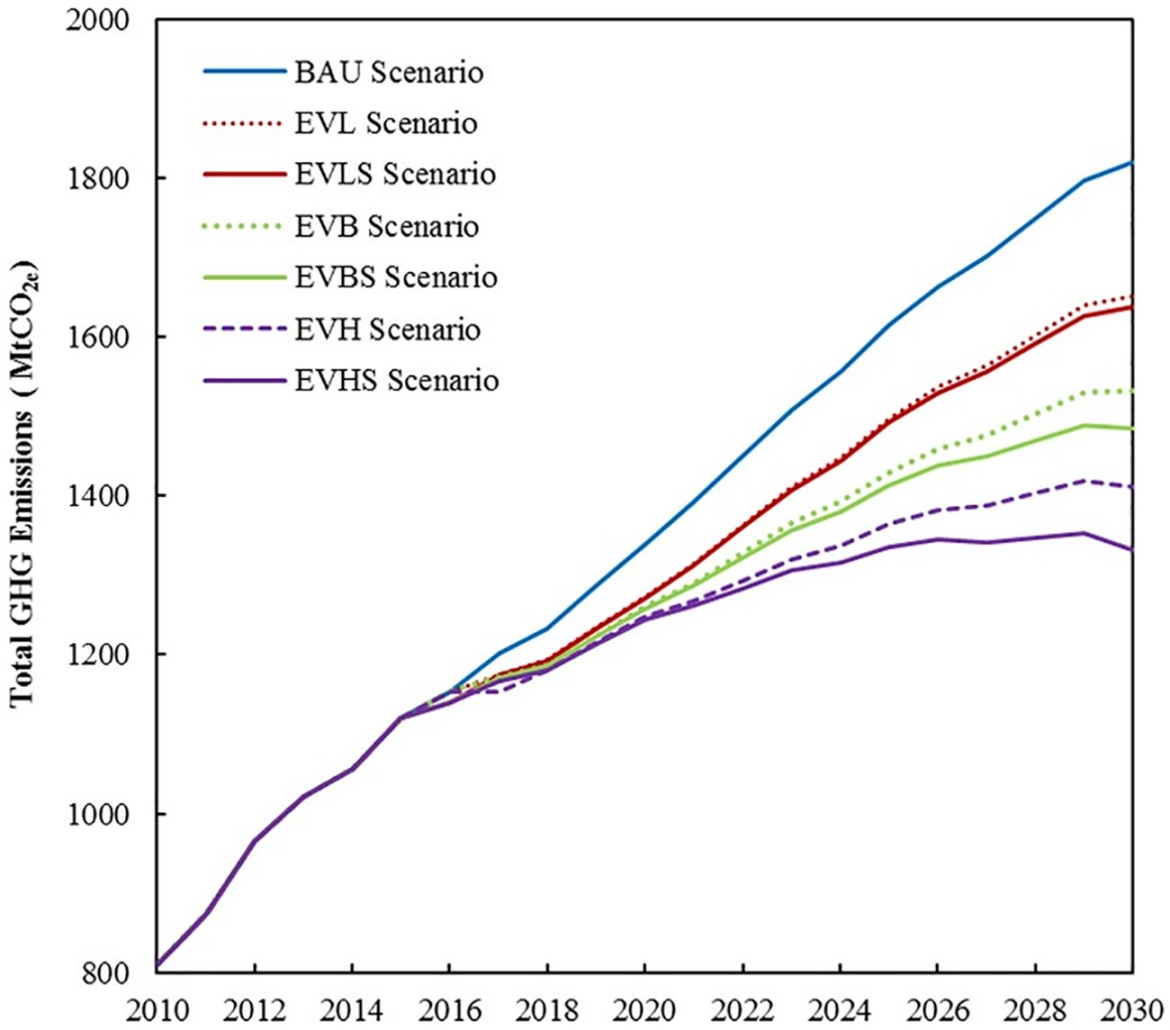
Total GHG emissions under various EV scenarios.

GHG emission reduction from the replacement of ICEVs with EVs (BEVs+PHEVs) under various EV penetration scenarios will reach 0.5%–2.3% and 3.0%–16.2%, respectively, in 2020 and 2030. Furthermore, GHG emission reduction from the replacement of ICEVs with CNGVs will be 62.55 MtCO_2e_ in 2020, which is a 4.7% reduction of the BAU scenario. In 2030, the GHG emission reduction will be 112.59 MtCO_2e_, which achieves a 6.19% emission reduction of the BAU scenario. Fig 10 depicts the total amount of GHG emissions under different EV replacement scenarios. In 2020, total GHG emissions will decrease by 6.9%, 5.9%, and 5.0% under the EVH, EVB, and EVL scenarios, respectively (including reduction from the replacement of ICEVs with CNGVs). However, total GHG emissions will decrease by 22.4%, 15.8%, and 9.2% under the three scenarios in 2030. In summary, BEVs will play a prominent role in terms of the mitigation of the effects of GHG emissions.

We also define three alternative scenarios of decarbonized electricity generation according to the IEA’s new policy scenario [26], namely, the EVHS, the EVBS, and the EVLS under which the share of coal-fired electricity generation will gradually be reduced to 60% and 55% in 2025 and 2030, respectively. The EVHS scenario combines the EVH scenario with the abovementioned decarbonized electricity generation. The same is true for the EVBS and EVLS scenarios (Table 4). The simulation results show that GHG emissions from the replacement of ICEVs with EVs will decrease by 20.6%, 12.2%, and 3.8% under the EVHS, EVBS, and EVLS scenarios, respectively, in 2030. Thereafter, the total GHG emission reduction will decrease by 26.8%, 18.4%, and 9.9% under the EVHS, EVBS, and EVLS scenarios, respectively, in 2030. The results confirm that if the replacement of ICEVs with EVs is accompanied by the decarbonization of the electricity generation mix to 55% coal-fired electricity in 2030, then total transport GHG emissions will be reduced by an additional of 0.8%–4.4%.

#### 5.3.3 GHG emission reduction from technological improvement

**Fig 11:**
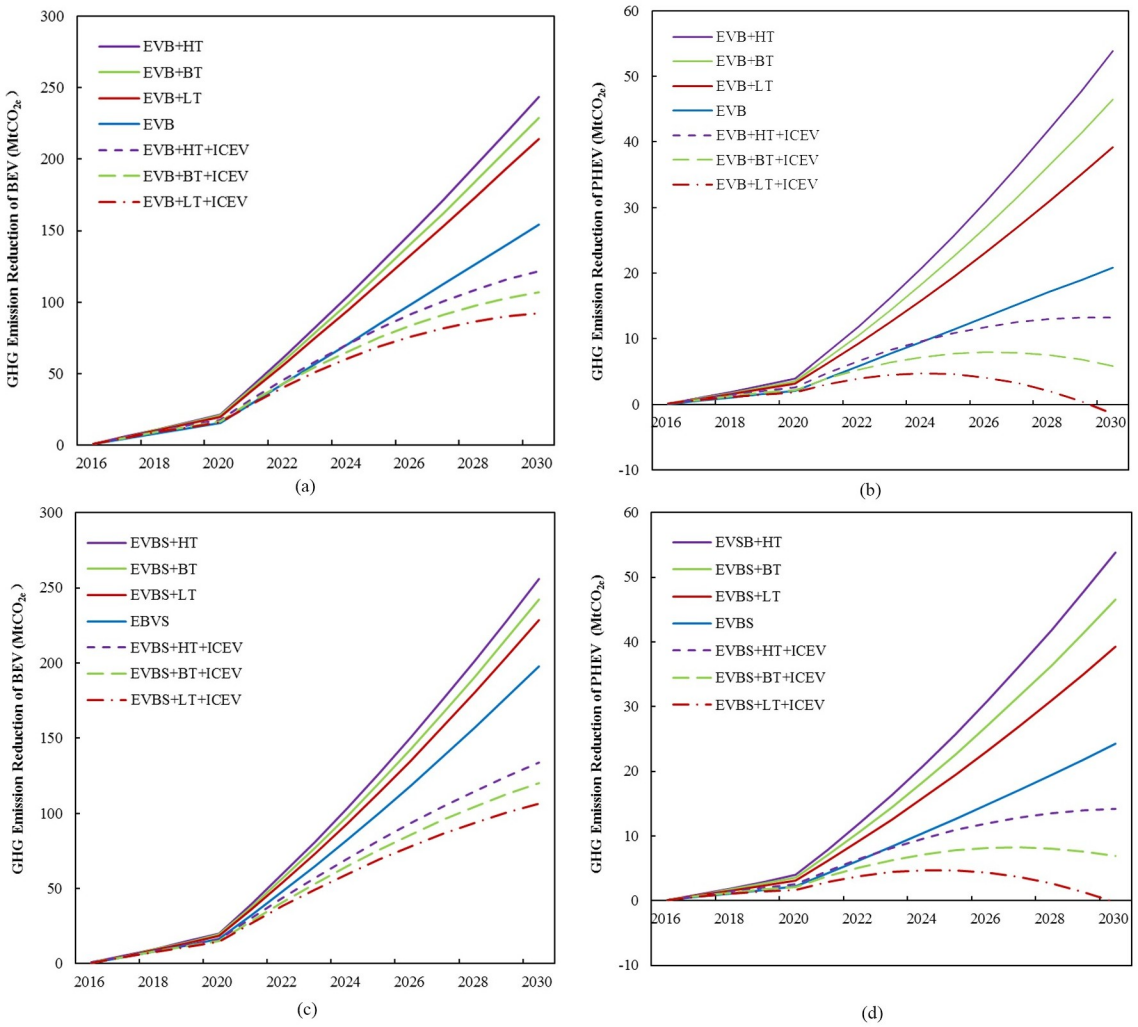
GHG emission reduction of BEVs and PHEVs. GHG emission reduction of (a) BEVs and (b) PHEVs under the current power generation mix and (c) BEVs and (d) PHEVs under the decarbonized power generation mix.

The simulation results of the reduction of GHG emissions from the replacement of ICEVs with EVs under the technological improvement scenarios of EVB+HT, EVB+BT, and EVB+LT are presented in Fig 11. The abovementioned three scenarios assume that EV ownership will increase to 47.5 million in 2030 (as assumed in the EVB scenario). However, the rate of technological improvement differs in each scenario. Moreover, Fig 11(a) and 11(b) show GHG emission reduction from the replacement of ICEVs with BEVs and PHEVs, respectively, provided that the power generation sector remains unchanged, with electricity generated by 68.2% coal. Fig 11(c) and 11(d) show GHG emission reduction under scenarios, with the decarbonized electricity generation mix using 60% and 55% coal in electricity generation in 2025 and 2030, respectively. In each figure, we compare emission reduction from the replacement of ICEVs with EVs under different technological improvement scenarios.

Under the EVB scenario with electricity generated by 68.2% coal, the replacement of ICEVs with BEVs and PHEVs will reduce GHG emissions in China’s transport sector by 154.06 MtCO_2e_ and 20.87 MtCO_2e_, respectively, in 2030 (see EVB in Fig 11(a) and 11(b)). The reduction of total GHG emissions is 9.6% of the BAU scenario. If we only consider the technological improvement of EVs without regarding that of ICEVs, then GHG emissions will be reduced by 13.9%–16.3% of the BAU scenario in 2030. Among them, GHG emissions from the replacement of ICEVs with BEVs and PHEVs will decrease by 228.84 MtCO_2e_ and 46.54 MtCO_2e_, respectively, in 2030 under the EVB+BT scenario (see Fig 11(a) and 11(b)). The reduction of GHG emissions from the replacement of ICEVs with EVs is 15.2% of the BAU scenario. An additional amount of GHG emissions of approximately 5.6% could be reduced with the technological improvement of EVs.

Fig 11(a)–11(d) illustrate the simulation results of the combined scenarios of EV technological improvement (e.g., EVB+HT, EVB+BT, and EVB+LT) with ICEV technological improvement (ICEV). Take the EVB+BT+ICEV scenario as an example. The emissions reduced by the replacement of ICEVs with BEVs will be 106.93 MtCO_2_ in 2030, which is a 69% of GHG emission reduction from BEVs under the EVB scenario. Meanwhile, emission reduction from the replacement of ICEVs with PHEVs under the EVB+BT+ ICEV scenario will be 5.90 MtCO_2e_ in 2030, which is approximately 28% of emission reduction from PHEV under the EVB scenario. In summary, GHG emission reduction from the replacement of ICEVs with EVs will be 6.2% of the BAU scenario. In addition, PHEVs will have no positive effect on the overall GHG emission reduction after 2030 under the EVB+LT+ICEV scenario.

If the decarbonization of the electricity sector can be carried out by reducing the share of coal in the electricity generation mix to 55% in 2030, then the replacement of ICEVs with BEVs and PHEVs will reduce GHG emissions by 197.98 MtCO_2e_ and 24.21 MtCO_2e_, respectively, under the EVBS scenario (see EVBS in Fig 11(c) and 11(d)). The GHG emission reduction accounts for 12.2% of emissions of the BAU scenario. The combined scenarios of the technological improvement of EVs and ICEVs are also investigated under the decarbonized electricity generation condition. Take the EVBS+BT+IECV scenario as an example. Emission reduction from the replacement of ICEVs with BEVs will be 120.09 MtCO_2e_ in 2030, which is approximately 61% of GHG emission reduction from BEVs of the EVBS scenario. Conversely, emission reduction from the replacement of ICEVs with PHEVs under the EVBS+BT+IECV scenario will be 6.90 MtCO_2e_ in 2030, which is approximately 29% of the GHG emission reduction of PHEVs of the EVBS scenario. We also confirm that even with aggressive electricity sector decarbonization, PHEVs only have marginal or negative effects on GHG emission reduction when ICEVs experience a dramatic technological improvement.

**Fig 12:**
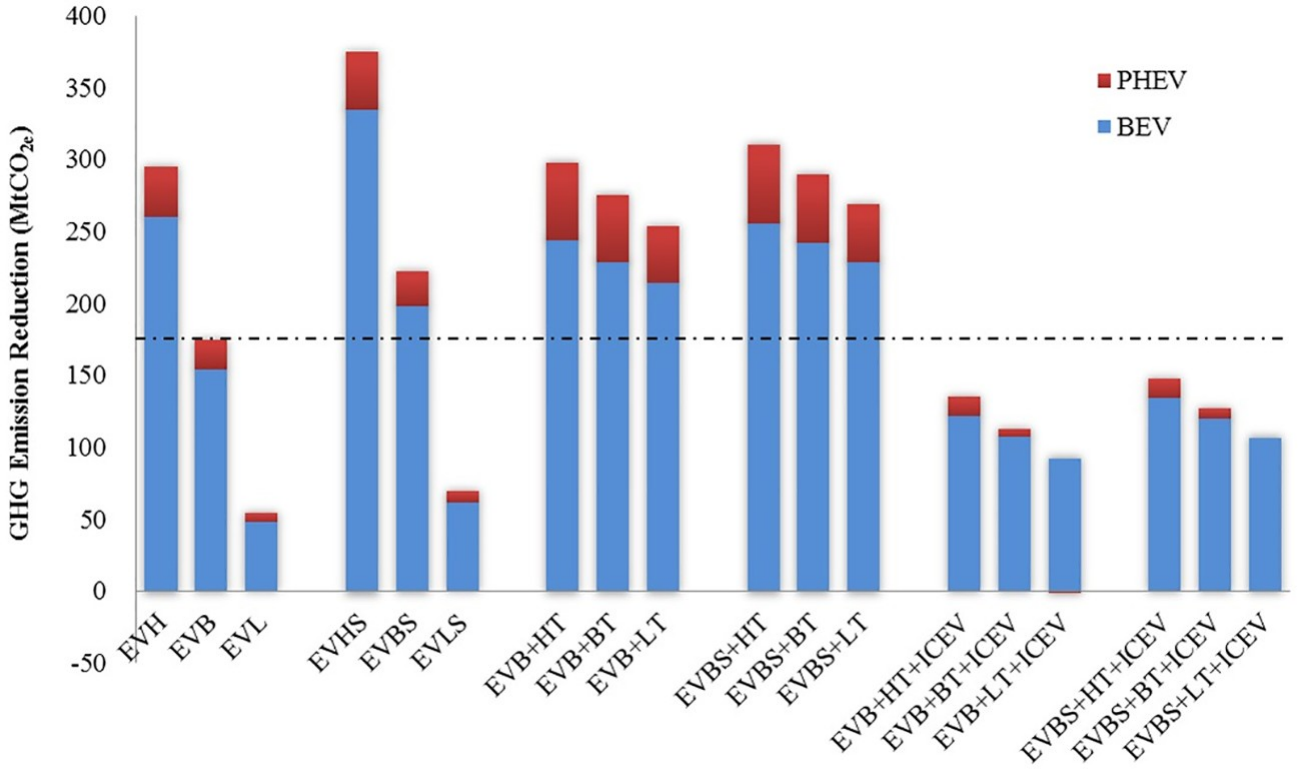
GHG emission reduction of EVs under various scenarios.

Fig 12 presents GHG emission reduction from the replacement of ICEVs with BEVs and PHEVs under various scenarios. The decarbonization of the power sector and the technological improvement of EVs are important factors that contribute to the additional reduction of GHG emissions. Meanwhile, the technological improvement of ICEVs should not be ignored. The simulation results in Fig 10 show that without further technological measures, the GHG emission reduction effect of EVs will be reduced by 35%–43% owing to the projected technological improvement of ICEVs. Policy makers in China should focus on the combining policies for technological improvement of both ICVEs and EVs. However, despite the limited room for fuel economy improvement of ICEVs, the technological improvement of ICEVs is effective in reducing GHG emissions in the short term [69]. The reduction of GHG emissions in the long term will increasingly rely on EVs in the transport sector.

### 5.4 Results comparison with other researches

Table 6 provides a comparative analysis between our results and those of published studies. Divergent results have been reported in the literature to discuss the use of EVs instead of ICEVs. However, previous studies have shown that the planning scenario of EVs will account for approximately 10% to 60% emission reduction in 2030. Research shows that areas with a high proportion of renewable energy in the electricity grid will generate increased energy-saving benefits. Such areas include France [7], Sweden [7] and California [35] in the United States. In addition, the differences in each study also reflect the following aspects. 1) Scenario parameters are set differently, which are mainly reflected in the differences in EV market share, electricity generation mix, technological improvement efficiency, and policy support for EV promotion. 2) The variability in methodological approaches for emission calculation leads to different evaluation ranges of the GHG emission reduction effect. For example, in Table 6, WTW refers to the research boundary covering only the life cycle of energy consumed by vehicles. WTW is subdivided into two stages, namely, well-to-tank (WTT) and tank-to-wheel (TTW). Complete LCA is a WTW with a cradle-to-grave life cycle of vehicles [70]. Wolfram & Wiedmann [33] observed that specific vehicle carbon footprints differ by up to 17% depending on whether life cycle analysis or hybrid life cycle is used. 3) The policy directions of EV promotion differ. For example, Watabe et al. [71] argued that high carbon taxes and accelerated infrastructure construction are required to promote the use of EVs in Japan; otherwise, consumers will tend to use fuel vehicles. Wolfram and Wiedmann [33] emphasized that policies for the electricity and vehicle markets should be regarded as inseparable. Furthermore, Dhar et al. [32] argued that policies to support the EV market are required on the supply and demand sides owing to India’s resource shortage in the domestic battery industry.

**Table 6:** Comparison between the present study and established literature.

Country or region	Scenario year	Method	Emission reduction considering the use of EVs instead of ICEVs	Scenario description	Reference
India	2030	WTW	−10% compared with BAU	Significant policy support for EV, technological improvement, and cost reduction	[32]
Australia	2030	Hybrid LCA	−32% compared with BAU	94% EV shares and 96% renewable energy shares compared with 18% EV shares and 36% renewable energy shares under BAU	[33]
America	2025	WTW	−65% in California and in the NPCC; –25% in Midwestern states.	California has 9% coal-based electricity; the three Midwestern states have 54%	[35]
Japan	2030	Complete LCA	−25.4% of the BAU scenario	Infrastructure development, high carbon tax, and high oil price	[71]
	2050	TTW and WTW	−86.9% of TTW and −69.6% of WTW compared with the baseline scenario	Market share of BEVs accounts for 99.5% of new vehicle sales in 2050	[72]
Greece (Athens City)	2020	WTW	−21% compared with baseline scenario	50% gasoline, 20% diesel, 10% electricity, 10% biodiesel, and 10% natural gas in the energy mix	[73]
Brail	2030	WTW	−30% emission reduction	Over 70% of electricity by hydroelectric power	[74]
China	2030	WTW	− (3.0~16.2)% compared with BAU	Projected EV penetration	Present study
			− (3.8%~20.6)% compared with BAU	Projected EV penetration with 55% coal-based electricity	
			−(5.8%~17.1)% compared with BAU	Projected EV penetration, 55% coal-based electricity, and EV technological improvement	

## 6 Conclusion and policy implications

This study investigates the potential reduction of GHG emissions in China’s transport sector. The CTEGER SD simulation model is established to simulate transport energy consumption and the reduction of GHG emissions from the replacement of ICEVs with EVs. The main conclusions are as follows. First, reductions in transport gasoline and diesel consumption by 4.4%–16.1% and 15.8%–34.3%, respectively, will be achieved by 2030 under China’s projected EV penetration scenarios. Second, GHG emission reduction from the replacement of ICEVs with EVs under various EV penetration scenarios will reach 3.0%–16.2% by 2030. Third, if the decarbonization of the electricity generation sector can be achieved by reducing coal-fired electricity generation to 55% in 2030, then total transport GHG emissions will be further reduced by 0.8%–4.4%. Fourth, under the combined technological improvement scenarios of EVs and ICEVs, GHG emission reduction will be approximately 5.8%–17.1% by 2030. This study shows that the replacement of ICEVs with EVs could generate a considerable effect on transport oil product consumption and on the reduction of GHG emissions. This scenario is possible despite the uncertainty in the influence intensity, which depends on the penetration rate of EVs, the decarbonization of the power sector, and the technological improvement of EVs and ICEVs.

EVs are satisfactory alternatives to ICEVs, especially in cities with serious air pollution issues. EVs should be actively and rationally developed to achieve ambitious development scenarios. The state must formulate policies to develop the related industrial chain of new EVs and strengthen the construction of relevant infrastructures given their cost disadvantage. Local policymakers can develop incentives for EVs by issuing highly strict emission standards, providing parking and toll fee discounts for EVs, and giving traffic priority for EVs. Policies that encourage the generation of considerably clean power in the electricity mix should also be enhanced. Moreover, policies for the electricity market and EVs should be combined. Policymakers should consider the additional demand of electricity from EVs and plan capacity expansions of renewable electricity to meet this need. If the charging of EVs is properly designed, then they may serve as distributed electricity storage capacities, which is beneficial for the balance of electricity supply–demand. Additional policies should focus on incentives for off-peak and time-division charging of EVs, which could further reduce the GHG emissions of electricity.

The results show that GHG emissions will be reduced by 14.0%–17.1% in 2030 if only the technological improvement of EVs is considered and that of ICEVs is ignored. However, under the combined technological improvement scenarios of EVs and ICEVs, the GHG emission reduction effect of EVs will be reduced by 35%–43% owing to the projected technological improvement of ICEVs. The results highlight the importance of the technological improvement of EVs to maximize GHG emission reductions. EVs will undergo substantial technological improvement in the long term and hold a promising future in terms of energy-saving efficiency and emission reduction. Nevertheless, room for ICEV technological improvement remains limited. The gap in GHG emissions between EVs and ICEVs is expected to widen. Thus, increasing investments in EV scientific research and guiding enterprises and research institutions to participate in the research and development of key technologies are necessary to increase the popularity of EVs.

## Supporting information

S1 Appendix The equations and data in CTEGER. (DOCX)
