# Supplementary material for: Transport oil product consumption and GHG emission reduction potential in China: An electric vehicle-based scenario analysis
Source: PLoS One. 2019 Sep 16;14(9):e0222448. doi: 10.1371/journal.pone.0222448 (PMC6746360; doi:10.1371/journal.pone.0222448)
Supplement: S1 Appendix — (DOCX) [file pone.0222448.s001.docx]

**Appendix**

The equations and data in the SD model are as follows:

1. INITIAL TIME = 1985
2. FINAL TIME = 2030
3. TIME STEP = 1
4. Units for Time = Year
5. GDP = INTEG (GDP Increment, 37614.2) Unit: Billion Yuan
6. Historical Value of GDP Growth Rate = ([(1985,0)-(2016,31)], (1985,8.8), (1986,11.6), ( 1987,11.3), (1988,4.1), (1989,3.8), (1990,9.2), (1991,14.2), (1992,13.9), (1993,13.1), (1994,10.9), (1995,10.0),(1996,9. 3), (1997,7.8), (1998,7.62), (1999,8.4), (2000,8.3), (2001,9.1), (2002,10.0), (2003,10.1), (2004,11.3), (2005,12.7), (2006,14.2), (2007,9.6), (2008,9.2), (2009,10.4), (2010,9.3), (2011,7.7), (2012,11.3), (2013,7.3), (2014,6.9), (2015,6.9), (2016,6.7)) Unit:%
7. GDP Increment = GDP* GDP Growth Rate /100 Unit: Billion Yuan
8. GDP Growth Rate = IF THEN ELSE (Time<2017, Historical Value of GDP Growth Rate, 6.9-0.15*(Time-2016)) Unit: %
9. GDP (t-1) = ACTIVEINITIAL (DELAY1 (GDP, 1), Initial Value=1) Unit: Billion Yuan
10. GDP (t-2) = ACTIVEINITIAL (DELAY1("GDP (t-1)", 1), Initial Value=1) Unit: Billion Yuan
11. Predicted Value of Transport Output = exp (IF THEN ELSE (Time <1990, 0, 0.93114*ln ("Transport Output Value (t-1)")+0.27829*ln (GDP) -0.223304*ln ("GDP (t-2)")))

Unit: Billion Yuan

1. Historical Value of Transport Output

=([(1985,0)-(2015,30000)], (1985,1884.93), (1986,2146.54), (1987,2352.76), (1988,2647.46), (1989,2758.59), (1990,2988.83), (1991,3304.33), (1992,3636.62), (1993,4092.65), (1994,4441.48), (1995,4929), (1996,5473.04), (1997,5976.62), (1998,6609.49), (1999,7414), (2000,8050.13), (2001,8759.14), (2002,9383.31), (2003,9958.16), (2004,11399.8), (2005,12676.9), (2006,13940.6), (2007,15585.8), (2008,16727.4), (2009,17425.9), (2010,19132.2), (2011,21020.5), (2012,22451.7), (2013,23294.2), (2014,24807), (2015,25954.9), (2016,26844.7)) Unit: Billion Yuan

1. Transport Output Value (t-1) = ACTIVEINITIAL (IF THEN ELSE (Time<2017, DELAY1 (Historical Value of Transport Output, 1) , DELAY1 (Predicted Value of Transport Output, 1) ), Initial Value=1) Unit: Billion Yuan
2. Predicted Value of Aeronautical Turnover = exp (IF THEN ELSE (Time<1990, 0, 5.83513+0.40634 *ln ("Aeronautical Turnover (t-1)") + 2.99636*ln (GDP) -2.17359*ln ("GDP (t-1)") Unit: Million People Kilometers
3. Historical Value of Aeronautical Turnover = ([(1985,0)-(2015,8000)], (1985,115.7), (1986,146.3), (1987,182.1), (1988,217), (1989,186.8), (1990,230.5), (1991,301.3), (1992,406.1), (1993,477.6), (1994,551.6), (1995,681.3), (1996,747.8), (1997,773.5), (1998,800.2), (1999,857.3), (2000,970.5), (2001,1091.4), (2002,1268.7), (2003,1263.2), (2004,1782.3), (2005,2044.9), (2006,2370.7), (2007,2791.7), (2008,2882.8), (2009,3375.2), (2010,4039), (2011,4537), (2012,5025.7), (2013,5656.8), (2014,6334.2), (2015,7282.6), (2016,8183.4))

Unit: Million People Kilometers

1. Aeronautical Turnover (t-1) = ACTIVEINITIAL (IF THEN ELSE (Time<2017, DELAY1 (Historical Value of Aeronautical Turnover,1), DELAY1 (Predicted Value of Aeronautical Turnover, 1) ), Initial Value=1) Unit: Million People Kilometers
2. Predicted Value of Urbanization Rate = 95/(1+0.365289*exp (-0.0677936*(Time-1984))) Unit: %
3. Historical Value of Urbanization Rate = ([(1985,70)-(2015,100)], (1985,71.56), (1986,72.86), (1987,73.19), (1988,74.3), (1989,74.89), (1990,72.88), (1991,75.47), (1992,78.21), (1993,80.29), (1994,80.14), (1995,80.04), (1996,80.31), (1997,81.71), (1998,82.44), (1999,83.53), (2000,84.94), (2001,85.61), (2002,86.26), (2003,87.2), (2004,86.61), (2005,87.88), (2006,88.89), (2007,89.23), (2008,89.27), (2009,89.67), (2010,89.9), (2011,89.9628), (2012,89.9179), (2013,89.9873), (2014,90.2026), (2015,90.4179) , (2016,90.6582)) Unit: %
4. Urbanization Rate (t-1) = ACTIVEINITIAL (IF THEN ELSE (Time<2017, DELAY1 (Historical Value of Urbanization Rate,1), DELAY1 (Predicted Value of Urbanization Rate,1)), Initial Value=1) Unit:%
5. Predicted Value of Private Car Ownership = (44550/(1+2317.06*exp (-0.226*(Time-1984)))

+32000/(1+2115.94*exp (-0.239*(Time-1984))))/2 Unit: Ten Thousand

1. Historical Value of Private Car Ownership = ([(1985,0)-(2015,20000)], (1985,28.49), (1986,34.71), (1987,42.29), (1988,60.42), (1989,73.12), (1990,81.62), (1991,96.04), (1992,118.2), (1993,155.77), (1994,205.42), (1995,249.96), (1996,289.67), (1997,358.36), (1998,423.65), (1999,533.88), (2000,625.33), (2001,770.777), (2002,968.98), (2003,1219.23), (2004,1481.66), (2005,1848.07), (2006,2333.32), (2007,2876.22), (2008,3501.39), (2009,4574.91), (2010,5938.71), (2011,7326.79), (2012,8838.6), (2013,10501.7), (2014,12339.4), (2015,14099.1), (2016,16456.5) ) Unit: Ten Thousand
2. Private Car Ownership (t-1) = ACTIVEINITIAL(IF THEN ELSE (Time<2017, DELAY1 (Historical Value of Private Car Ownership,1), DELAY1 (Predicted Value of Private Car Ownership,1)), Initial Value=1) Unit: Ten Thousand
3. Predicted Value of Energy Consumption Structure = IF THEN ELSE (Time<2016, 1, IF THEN ELSE (Time<2021, 63-Time+2015, 58-(58-42)/15*(Time-2020))) Unit: %
4. Historical Value of Energy Consumption Structure = ([(1985,60)-(2015,80)], (1985,75.8), (1986,75.8), (1987,76.2), (1988,76.2), (1989,76), (1990,76.2), (1991,76.1), (1992,75.7), (1993,74.7), (1994,75), (1995,74.6), (1996,73.5), (1997,71.4), (1998,70.9), (1999,70.6), (2000,68), (2001,68.5), (2002,68.5), (2003,70.2), (2004,70.2), (2005,72.4), (2006,72.5), (2007,71.5), (2008,71.5), (2009,71.6), (2010,69.2), (2011,70.2), (2012,68.5), (2013,67.4), (2014,65.6), (2015,64), (2016,68)) Unit: %
5. Energy Consumption Structure (t-1) = ACTIVEINITIAL (IF THEN ELSE (Time <2017, DELAY1 (Historical Value of Energy Consumption Structure,1), DELAY1 (Predicted Value of Energy Consumption Structure,1)), Initial Value=1) Unit: %
6. Predicted Value of Energy Consumption Intensity = ([(1990,0)-(2030,2)], (1990,1.83995), (1991,1.73554), (1992,1.65498), (1993,1.4645), (1994,1.41074), (1995,1.31809), (1996,1.30784), (1997,1.17436), (1998, 1.07232), (1999,1.01536), (2000,1.0026), (2001, 0.958861), (2002, 0.948381), (2003,0.964946), (2004,1.07038), (2005, 1.11478), (2006, 1.08713), (2007, 1.02363), (2008, 0.972883), (2009, 0.908286), (2010, 0.898035), (2011, 0.875075), (2012, 0.864478), (2013,0.815501),(2014, 0.737027), (2015, 0.729055), (2016, 0.702), (2017, 0.672), (2018, 0.651), (2019,0.629), (2020, 0.605), (2021, 0.587), (2022, 0.572), (2023, 0.551), (2024, 0.535), (2025,0.517), (2026, 0.493), (2027, 0.475), (2028, 0.463), (2029, 0.445), (2030, 0.433))

Unit: Tons of Standard Coal/ Ten Thousand Yuan

1. Historical Value of Energy Consumption Intensity = ([(1985,0)-(2016,3)], (1985, 2.03864), (1986,1.97476), (1987,1.89633), (1988,1.8293), (1989,1.83229), (1990,1.79675), (1991,1.73039), (1992,1.59331), (1993,1.48546), (1994,1.39), (1995,1.33926), (1996,1.25469), (1997,1.15405), (1998,1.07238), (1999,1.02854), (2000,0.991717), (2001,0.969189), (2002,0.968636), (2003,1.02317), (2004,1.086), (2005,1.10737), (2006,1.07716), (2007,1.02579), (2008,0.96319), (2009,0.924606), (2010,0.898223), (2011,0.881942), (2012,0.8512), (2013,0.792858), (2014,0.754715), (2015,0.71285) , (2016,0.70245))

Unit: Tons of Standard Coal/ Ten Thousand Yuan

1. Energy Consumption Intensity (t-1) = ACTIVEINITIAL(IF THEN ELSE (Time < 2017,

DELAY1 (Historical Value of Energy Consumption Intensity, 1), DELAY1 (Predicted Value of Energy Consumption Intensity, 1)), Initial Value=1)

Unit: Tons of Standard Coal/ Ten Thousand Yuan

1. The Density of Gasoline = 0.725 Unit：kg/L
2. The Density of Diesel = 0.825 Unit：kg/L
3. The Density of Kerosene = 0.775 Unit：kg/L
4. Predicted Value of Transport Gasoline Consumption = IF THEN ELSE (Time<1991, 0, exp (ln ("Transport Gasoline Consumption (t-1)") -0.064797*(ln ("Transport Gasoline Consumption (t-1)")-4.65317 *ln ("Urbanization Rate (t-1)") -0.654507* ln ("Private Car Ownership (t-1)")

-3.83573*ln ("Energy Consumption Intensity (t-1)")+17.4977) - 0.264299 * (DELAY1(ln ("Transport Gasoline Consumption (t-1)")-ln (DELAY1("Transport Gasoline Consumption (t-1)",1)),2)) -2.25878*(ln ("Urbanization Rate (t-1)") –ln (DELAY1("Urbanization Rate (t-1)",1))) -0.930391*(DELAY1(ln ("Urbanization Rate (t-1)") -ln(DELAY1 ("Urbanization Rate (t-1)",1)),1)) +0.725882*(ln ("Energy Consumption Intensity (t-1)") -ln(DELAY1 ("Energy Consumption Intensity (t-1)",1))) -0.975213*(DELAY1(ln ("Energy Consumption Intensity (t-1)")-ln(DELAY1 ("Energy Consumption Intensity (t-1)",1)),1)) +0.657888*(DELAY1 (ln ("Energy Consumption Intensity (t-1)")-ln (DELAY1 ("Energy Consumption Intensity (t-1)",1)),2)) +0.41912*(DELAY1 (ln ("Private Car Ownership (t-1)")-ln (DELAY1 ("Private Car ownership (t-1)",1)),2)) +0.21839))

Unit: Ten Thousand Tons

1. Historical Value of Transport Gasoline Consumption = ([(1985,0)-(2016,9000)], (1985,1108.85), (1986,1171.11), (1987,1257.32), (1988,1350.52), (1989,1353.86), (1990,1428.86), (1991,1601.4), (1992,1766.89), (1993,1890.92), (1994,1919.9), (1995,2095.91), (1996,2209.72), (1997,2242.93)，(1998,2246.83), (1999,2287.05), (2000,2381.54), (2001,2442.69), (2002,2571.47), (2003,2959.85), (2004,3445.83), (2005,3604.03), (2006,3921.82), (2007,4179.07), (2008,4849.19), (2009,4909.9), (2010,5503.6), (2011,5861.26), (2012,6436.94), (2013,7284.32), (2014,7779.51), (2015,8972.41), (2016,9446.34))

Unit: Ten Thousand Tons

1. Transport Gasoline Consumption (t-1)= ACTIVEINITIAL(IF THEN ELSE (Time<2017, DELAY1(Historical Value of Transport Gasoline Consumption,1) , DELAY1 (Predicted Value of Transport Gasoline Consumption,1)), Initial Value=1) Unit: Ten Thousand Tons
2. GHG Emissions of Transport Gasoline Consumption= IF THEN ELSE (Time<2016, Historical Value of Transport Gasoline Consumption*GHG Emission Coefficient of Gasoline/The Density of Gasoline , Predicted Value of Transport Gasoline Consumption*GHG Emission Coefficient of Gasoline/The Density of Gasoline )/1000 Unit: Ten Thousand Tons of CO_2e_
3. Predicted Value of Transport Diesel Consumption = IF THEN ELSE (Time<1990, 0, exp (ln ("Transport Diesel Consumption (t-1)")-0.079257*(ln ("Transport Diesel Consumption (t-1)")-0.87747*ln ("Energy Consumption Intensity (t-1)") -1.29784*ln ("Energy Consumption Structure (t-1)")-1.28319*ln ("Transport Output Value (t-1)")+8.75399)

+0.45224*(ln ("Transport Diesel Consumption (t-1)") – ln (DELAY1 ("Transport Diesel Consumption (t-1)",1))) +0.285051*(DELAY1 (ln ("Transport Diesel Consumption (t-1)") –ln (DELAY1 ("Transport Diesel Consumption (t-1)",1)),2)) -0.412572* (DELAY1 (ln ("Energy Consumption Intensity (t-1)")-ln (DELAY1 ("Energy Consumption Intensity (t-1)",1)),2)) -0.404335*(DELAY1 (ln ("Transport Output Value (t-1)")-ln(DELAY1 ("Transport Output Value (t-1)", 1)), 2))+ 1.16709*(ln ("Energy Consumption Structure (t-1)")-ln (DELAY1 ("Energy Consumption Structure (t-1)",1)))+0.047164)) Unit: Ten Thousand Tons

1. Historical Value of Transport Diesel Consumption = ([(1985,0)-(2015,20000)], (1985,1354.49), (1986,1528.95), (1987,1673.52), (1988,1778.51), (1989,1877.99), (1990,1892.47), (1991,1976.81), (1992,2140.43), (1993,2263.49), (1994,2365.83), (1995,2721.1), (1996,2830.77), (1997,3149.18), (1998,3588.62), (1999,4122.73), (2000,4546.21), (2001,4773.08), (2002,5223.26), (2003,5790.46), (2004,6882.81), (2005,7853.9), (2006,8711), (2007,9619.56), (2008,10321.2), (2009,10613.8), (2010,11386.4), (2011,12427.1), (2012,13771.4), (2013,14076.4), (2014,14221.5), (2015,14322), (2016,14549)) Unit: Ten Thousand Tons
2. Transport Diesel Consumption (t-1) = ACTIVEINITIAL (IF THEN ELSE (Time<2017, DELAY1(Historical Value of Transport Diesel Consumption, 1) , DELAY1 (Predicted Value of Transport Diesel Consumption, 1)), Initial Value=1) Unit: Ten Thousand Tons
3. GHG Emissions of Transport Diesel Consumption = IF THEN ELSE (Time<2016, Historical Value of Transport Diesel Consumption*Emission Coefficient of Diesel/The Density of Diesel , Predicted Value of Transport Diesel Consumption*GHG Emission Coefficient of Diesel/The Density of Diesel )/1000

Unit: Ten Thousand Tons CO_2e_

1. Predicted Value of Transport Kerosene Consumption = IF THEN ELSE (Time< 1990, 0, exp (ln ("Transport Kerosene Consumption (t-1)")-0.154492*(ln ("Transport Kerosene Consumption (t-1)")-1.32151*ln ("Aeronautical Turnover (t-1)")-2.3874* ln ("Energy Consumption Intensity (t-1)")+3.42222)-0.206979*(ln ("Transport Kerosene Consumption (t-1)") –ln (DELAY1 ("Transport Kerosene Consumption (t-1)",1)))+0.200283*(ln ("Aeronautical Turnover (t-1)") –ln (DELAY1 ("Aeronautical Turnover(t-1)",1)))-0.420068*(ln ("Energy Consumption Intensity (t-1)")- ln(DELAY1 ("Energy Consumption Intensity (t-1)",1))) +0.204366) )

Unit: Ten Thousand Tons

1. Historical Value of Transport Kerosene Consumption = [(1985,0)-(2015,3000)], (1985,56.2), (1986,53), (1987,67.7), (1988,68.6), (1989,72.2), (1990,93.4), (1991,132.2), (1992,165), (1993,182.505), (1994,200.01),(1995,250.01), (1996,298.86), (1997,420.1), (1998,390.48), (1999,505.62), (2000,536.4), (2001,560.69), (2002,616.74), (2003,621.68), (2004,819.71), (2005,882.421), (2006,1000.54), (2007,1129.98), (2008,1174.59), (2009,1314.25), (2010,1601.08), (2011,1646.35), (2012,1787.09), (2013,1998.18), (2014,2216.03), (2015,2504.88), (2016,2798.75) )

Unit: Ten Thousand Tons

1. Transport Kerosene Consumption (t-1)= ACTIVEINITIAL (IF THEN ELSE (Time<2017, DELAY1 (Historical Value of Transport Kerosene Consumption,1) , DELAY1 (Predicted Value of Transport Kerosene Consumption,1)), Initial Value=1) Unit: Ten Thousand Tons
2. GHG Emissions of Transport Kerosene Consumption = IF THEN ELSE (Time<2016, Historical Value of Transport Kerosene Consumption*GHG Emission Coefficient of Kerosene/The Density of Kerosene, Predicted Value of Transport Kerosene Consumption* GHG Emission Coefficient of Kerosene/The Density of Kerosene )/1000 Unit: Ten Thousand Tons of CO_2e_
3. GHG Emission Intensity of Transport Oil Products = GHG Emissions of Transport Oil Products Consumption / Predicted Value of Transport Output Unit: Ton CO_2e_/Ten Thousand Yuan
4. New Ownership of BEV = New Ownership of EV *0.75 Unit: Ten Thousand
5. New Ownership of BEV Private Car = New Ownership of BEV *43/50 Unit: Ten Thousand
6. New Ownership of BEV Taxi= New Ownership of BEV *3/50 Unit: Ten Thousand
7. New Ownership of BEV Bus = New Ownership of BEV *4/50 Unit: Ten Thousand
8. New Ownership of PHEV = New Ownership of EV *0.25 Unit: Ten Thousand
9. New Ownership of PHEV Private Car = New Ownership of PHEV *43/50 Unit: Ten Thousand
10. New Ownership of PHEV Taxi = New Ownership of PHEV *3/50 Unit: Ten Thousand
11. New Ownership of PHEV Bus = New Ownership of PHEV *4/50 Unit: Ten Thousand
12. New Ownership of CNGV = IF THEN ELSE (Time<2016, 0 , IF THEN ELSE (Time<2021, 500+(1000-500)/5*(Time-2015)-500 , 1000+(1400-1000)/10*(Time-2020)-500 ) )

Unit: Ten Thousand

1. New Ownership of CNGV Taxi = New Ownership of CNGV *0.67 Unit: Ten Thousand
2. New Ownership of CNGV Bus = New Ownership of CNGV *0.33 Unit: Ten Thousand
3. Share of Thermal Power Generation = ([(2015,50)-(2030,80)], (2015,74.2363), (2016,68.2), (2017,67.26), (2018,66.31), (2019,65.37), (2020,64.43), (2021,63.49), (2022,62.54), (2023,61.6), (2024,60.66), (2025,59.71), (2026,58.77), (2027,57.83), (2028,56.89), (2029,55.94), (2030,55)) Unit: %
4. Share of Hydroelectric Power Generation = ([(2015,0)-(2030,20)], (2015,19.5853), (2016,19.4), (2017,19.23), (2018,19.06), (2019,18.89), (2020,18.71), (2021,18.54), (2022,18.37), (2023,18.2), (2024,18.03), (2025,17.86), (2026,17.69), (2027,17.51), (2028,17.34), (2029,17.17), (2030,17)) Unit: %
5. Share of Nuclear Power Generation = ([(2016,3)-(2030,11)], (2016,3.5), (2017,3.96), (2018,3.43), (2019,4.89), (2020,5.36), (2021,5.82), (2022,6.29), (2023,6.75), (2024,7.21), (2025,7.68), (2026,8.14), (2027,8.61), (2028,9.07), (2029,9.54), (2030,10)) Unit: %
6. Share of Wind Power Generation = ([(2016,3)-(2030,11)], (2016,3.9), (2017,4.34), (2018,4.77), (2019,5.21), (2020,5.64), (2021,6.08), (2022,6.51), (2023,6.95), (2024,7.39), (2025,7.82), (2026,8.26), (2027,8.69), (2028,9.13), (2029,9.56), (2030,10)) Unit: %
7. Share of Natural Gas Power Generation = 4 Unit: %
8. Share of PV Power Generation = ([(2016,0)-(2030,5)],(2016,1), (2017,1.21), (2018,1.43), (2019,1.64), (2020,1.86), (2021,2.07), (2022,2.29), (2023,2.5), (2024,2.71),

(2025,2.93), (2026,3.14), (2027,3.36), (2028,3.57), (2029,3.79), (2030,4)) Unit: %

1. Electricity Consumption per Mileage of BEV Private Car = ([(2015,15)-(2030,19)], (2015,18.73), (2016,17.33), (2017,17.154), (2018,16.978), (2019,16.8), (2020,16.626), (2021,16.45),

(2022,16.274), (2023,16.098), (2024,15.922),(2025,15.746),(2026,15.57),(2027,15.394),

(2028,15.218), (2029,15.042), (2030,14.866)) Unit: kWh/100 km

1. Electricity Consumption per Mileage of BEV Bus = ([(2015,80)-(2030,100)], (2015,98), (2016,90.65), (2017,89.727), (2018,88.804), (2019,87.881), (2020,86.958), (2021,86.035), (2022,85.112), (2023,84.189), (2024,83.266), (2025,82.343), (2026,81.42), (2027,80.497), (2028,79.574), (2029,78.651), (2030,77.73)) Unit: kWh/100 km
2. Gasoline Consumption per mileage of PHEV Private Car = ([(2015,2)-(2030,5)], (2015,4.25), (2016,3.93), (2017,3.89), (2018,3.85), (2019,3.81), (2020,3.77), (2021,3.73), (2022,3.69), (2023,3.65), (2024,3.61), (2025,3.57), (2026,3.53), (2027,3.49), (2028,3.45), (2029,3.41),(2030,3.37)) Unit: L/100 km
3. Electricity Consumption per Mileage of PHEV Private Car = ([(2015,7)-(2030,10)], (2015,9), (2016,8.33), (2017,8.245), (2018,8.16), (2019,8.075), (2020,7.99), (2021,7.905), (2022,7.82), (2023,7.735), (2024,7.65), (2025,7.565), (2026,7.48), (2027,7.395), (2028,7.31),(2029,7.225), (2030,7.14)) Unit: kWh/100 km
4. Diesel Consumption per Mileage of PHEV Bus = ([(2015,20)-(2030,50)], (2015,30), (2016,27.75), (2017,27.468), (2018,27.186), (2019,26.904), (2020,26.622), (2021,26.34), (2022,26.058), (2023,25.776), (2024,25.494), (2025,25.212), (2026,24.93), (2027,24.65), (2028,24.366), (2029,24.084), (2030,23.8)) Unit: L/100 km
5. Natural Gas Consumption per Mileage of CNGV Private Car = 9 Unit: m^3^/100 km
6. Natural Gas Consumption per Mileage of CNGV Bus = 37.5 Unit: m^3^/100 km
7. GHG Emission Coefficient of Thermal Power =1133.12 Unit: gCO_2e_ /kWh
8. GHG Emission Coefficient of Hydroelectric Power = 17.07 Unit: gCO_2e_ /kWh
9. GHG Emission Coefficient of Nuclear Power = 9.05 Unit: gCO_2e_ /kWh
10. GHG Emission Coefficient of Wind Power = 31.98 Unit: gCO_2e_ /kWh
11. GHG Emission Coefficient of Natural Gas Power = 773.34 Unit: gCO_2e_ /kWh
12. GHG Emission Coefficient of PV Power = 140 Unit: gCO_2e_ /kWh
13. GHG Emission Coefficient of Power Generation = (GHG Emission Coefficient of Nuclear Power *Share of Nuclear Power Generation+ GHG Emission Coefficient of Hydroelectric Power * Share of Hydroelectric Power Generation + GHG Coefficient of Thermal Power * Share of Thermal Power Generation + GHG Emission Coefficient of Wind Power * Share of Wind Power Generation + GHG Emission Coefficient of PV Power * Share of PV Power Generation + GHG Emission Coefficient of Natural Gas Power* Share of Natural Gas Power Generation)/100

Unit: gCO_2e_ /kWh

1. GHG Emission Coefficient of Gasoline = 2781.57 Unit：gCO_2e_/L
2. GHG Emission Coefficient of Diesel = 3827.94 Unit：gCO_2e_/L
3. GHG Emission Coefficient of Kerosene = 3448.8 Unit：gCO_2e_/L
4. GHG Emission Coefficient of Natural Gas = 2220.84 Unit：gCO_2e_/L
5. Gasoline Consumption per Mileage of Gasoline Car = ([(2015,0)-(2030,10)], (2015,7.82), (2016,7.665), (2017,7.51), (2018,7.356), (2019,7.2013), (2020,7.047), (2021,6.892), (2022,6.737), (2023,6.583), (2024,6.428), (2025,6.273), (2026,6.1187), (2027,5.964), (2028,5.809), (2029,5.655), (2030,5.5)) Unit: L/100 km
6. Diesel Consumption per Mileage of Diesel Bus = [(2015,0)-(2030,30)], (2015,36.39), (2016,35.671), (2017,34.952), (2018,34.233), (2019,33.514), (2020,32.795), (2021,32.076), (2022,31.357), (2023,30.638), (2024,29.919), (2025,29.2), (2026,28.481), (2027,27.762), (2028,27.043), (2029,26.324), (2030,25.6)) Unit: L/100 km
7. GHG Emissions per Mileage of Gasoline Car = GHG Emission Coefficient of Gasoline* Gasoline Consumption per Mileage of Gasoline Car Unit: gCO_2e_/100 km
8. GHG Emissions per Mileage of Diesel Bus = GHG Emission Coefficient of Diesel*Diesel Consumption per Mileage of Diesel Bus Unit: gCO_2e_/100 km
9. GHG Emissions per Mileage of BEV Private Car = Electricity Consumption per Mileage of BEV Private Car*GHG Emission coefficient of Power Generation Unit: gCO_2e_/100 km
10. GHG Emissions per Mileage of BEV Bus = Electricity Consumption per Mileage of BEV Bus* GHG Emission Coefficient of Power Generation Unit: gCO_2e_/100 km
11. GHG Emissions per mileage of PHEV Private Car = GHG Emission Coefficient of Gasoline *Gasoline Consumption per Mileage of PHEV Private Car + GHG Emission Coefficient of Power Generation*Electricity Consumption per Mileage of PHEV Private Car Unit: gCO_2e_/100 km
12. GHG Emissions per Mileage of PHEV Bus = GHG Emission Coefficient of Diesel*Diesel Consumption per mileage of PHEV Bus + GHG Emission Coefficient of Power Generation*Electricity Consumption per Mileage of PHEV Bus Unit: gCO_2e_/100 km
13. GHG Emissions per Mileage of CNGV Private Car = GHG Emission Coefficient of Natural Gas*Natural Gas Consumption per Mileage of CNGV Private Car Unit: gCO_2e_/100 km
14. GHG Emissions per Mileage of CNGV Bus = GHG Emission Coefficient of Natural Gas*Natural Gas Consumption per Mileage of CNGV Bus Unit: gCO_2e_/100 km
15. Mileage of Private Car = 180 Unit: 100 km
16. Mileage of Taxi = 1200 Unit: 100 km
17. Mileage of Bus = 600 Unit: 100 km
18. GHG Emission Reduction of BEV = (New Ownership of BEV Bus*( GHG Emissions per Mileage of Diesel Bus- GHG Emissions per Mileage of BEV Bus)*Mileage of Bus + New Ownership of BEV Taxi*( GHG Emissions per Mileage of Gasoline Car- GHG Emission per Mileage of BEV Private Car)*Mileage of Taxi + New Ownership of BEV Private Car*( GHG Emissions per Mileage of Gasoline Car- GHG Emissions per Mileage of BEV Private Car)*Mileage of Private Car)/1e+006 Unit: Ten Thousand Tons of CO_2e_
19. GHG Emission Reduction of PHEV = (New Ownership of PHEV Bus*( GHG Emissions per Mileage of Diesel Bus- GHG Emissions per Mileage of PHEV Bus)*Mileage of Bus+ New Ownership of PHEV Taxi*( GHG Emissions per Mileage of Gasoline Car- GHG Emissions per Mileage of PHEV Private Car)*Mileage of Taxi+ New Ownership of PHEV Private Car*( GHG Emissions per Mileage of Gasoline Car- GHG Emissions per Mileage of PHEV Private Car)*Mileage of Private Car)/1e+006 Unit: Ten Thousand Tons of CO_2e_
20. GHG Emission Reduction of CNGV = (New Ownership of CNGV Bus*( GHG Emissions per Mileage of Diesel Bus- GHG Emissions per Mileage of CNGV Bus)*Mileage of Bus + New Ownership of CNGV Taxi*( GHG Emissions per Mileage of Gasoline Car- GHG Emissions per Mileage of CNGV Private Car)*Mileage of Taxi)/1e+006 Unit: Ten Thousand Tons of CO_2e_
